# Structural Elucidation and Antiviral Properties of Pannosides from the Halophyte *Aster tripolium* L.

**DOI:** 10.3390/md22120524

**Published:** 2024-11-21

**Authors:** Jaeyoun Lee, Jae-Hyoung Song, Seo-Hyeon Mun, Hyun-Jeong Ko, Soohyun Um, Seung Hyun Kim

**Affiliations:** 1 College of Pharmacy, Yonsei Institute of Pharmaceutical Sciences, Yonsei University, Incheon 21983, Republic of Korea; jaeyoun1024@yonsei.ac.kr; 2 Department of Pharmacy, Kangwon National University, Chuncheon 24341, Republic of Korea; thdwohud@naver.com (J.-H.S.); moonnari0606@gmail.com (S.-H.M.); hjko@kangwon.ac.kr (H.-J.K.)

**Keywords:** halophytes, *Aster tripolium* L., terpenoid saponins, antiviral activities

## Abstract

Four previously undescribed pentacyclic triterpenoid saponins, pannosides F–I (**1**–**4**), were isolated from the halophyte *Aster tripolium* L. (*Tripolium pannonicum*), and their chemical structures were elucidated using 1D and 2D NMR spectroscopy and mass spectrometry. Comprehensive structural analysis revealed the presence of distinct aglycone and glycosidic moieties, along with complex acylation patterns. The acyl chains of pannosides, 3-hydroxybutyrate (3-HB) residues, were derivatized with (*S*)- and (*R*)- phenylglycine methyl ester to resolve the absolute configurations of the chiral centers in 3-HB. Then, the acyl chain-containing saponins, pannosides were evaluated for their antiviral activities against enterovirus A71 (EV71), coxsackievirus B3 (CVB3), and rhinovirus 1B (HRV1B). Pannosides exhibited antiviral activities against HRV1B, EV71, and CVB3. These findings suggest that saponins from *A. tripolium* exhibit potential antiviral activities and could be further explored for their therapeutic applications.

## 1. Introduction

Halophytes are plants that live in salty environments such as tidal flats and coastal areas. They have developed unique physiological and biochemical adaptations to thrive in such hostile environments [1]. These adaptations typically include the development of a variety of secondary metabolites to protect against environmental stresses such as oxidative stress and excessive salt [2]. The secondary metabolites of halophytes can also serve as biologically active molecules in therapeutic applications. For example, *Atriplex halimus* produces phenolic compounds that are antioxidants and anti-inflammatory [3]. *Salicornia europaea* also contains flavonoids, tannins, and sterols that have antibacterial and antidiabetic effects [4,5,6].

Among the various bioactive substances, saponins have received the most attention because of their potential for human treatment and their protective function in plants. For example, halophytes such as *Salicornia* and *Atriplex* are well known for producing large amounts of saponin, which aids in their survival in salty environments and may have therapeutic applications [7,8,9]. Additionally, the medicinal potential of saponins has been investigated in *Crithmum maritimum* (sea fennel), with the *Kochia* species being used in veterinary medicine and ethno-pharmacology [10]. The anticancer effects of *Aster tripolium* L., which has a high saponin content, demonstrate the potential of halophytes as a significant reserve for the discovery of new biologically active compounds with special qualities that set them apart from non-halophytes [11]. In our previous study on the anticancer effects, pannosides A–E (**5**–**9**) reduced the viability of cancer cell lines such as HT29 (colorectal adenocarcinoma cell lines), PC3 (prostate carcinoma cell lines), and PC9 (non-small cell lung carcinoma cell lines) [12].

The need for new antiviral agents is highlighted by the increasing incidence of viral diseases and the shortcomings of existing antiviral therapies [13]. Drug discovery has always relied heavily on natural products because of their structural diversity [14]. In addition to their well-known anti-inflammatory, antioxidant, and anticancer effects, saponins have shown encouraging antiviral activity. For example, ginsenoside Rd interferes with the life cycle of coxsackievirus B3 (CVB3), whereas ginsenoside Rb1 inhibits enterovirus 71 (EV71) by blocking viral replication [15]. Similarly, glycyrrhizin, a saponin from licorice root (*Glycyrrhiza glabra*), suppresses hepatitis C virus and influenza A virus (IAV) [16,17,18]. In another study, a fraction rich in the acylated triterpene saponins, QS-17, QS-18, and QS-21 from *Quillaja saponaria*, showed the prevention of rotavirus causing severe diarrhea [19]. On the basis of these studies, we aimed to investigate the structure and potential antiviral properties of four previously unreported saponins with unusual poly 3-HB chains, pannosides F–I (**1**–**4**), isolated from *A. tripolium*, in addition to pannosides A–E (**5**–**9**). In this study, we evaluated the antiviral activities of all pannosides (**1**–**9**) against enterovirus A71 (EV71), coxsackievirus B3 (CVB3), and rhinovirus 1B (HRV1B).

## 2. Results and Discussion

Pannoside F (**1**) was obtained as a white amorphous powder, and the molecular formula was identified as C_67_H_102_O_31_, with an exact mass of *m*/*z* 1425.6296 [M + Na]^+^ (calcd. for C_67_H_102_O_31_Na, *m*/*z* 1425.6297), as confirmed by high-resolution mass spectrometry (HRMS). The structure elucidation was carried out by using 1D and 2D nuclear magnetic resonance (NMR) techniques, including correlation spectroscopy (COSY), heteronuclear single quantum coherence (HSQC), heteronuclear multiple bond correlation (HMBC), and rotating-frame Overhauser effect spectroscopy (ROESY), along with infrared (IR) spectroscopy and mass spectrometry (MS). The ^1^H NMR spectrum of pannoside F showed characteristic signals corresponding to both the aglycone and the glycosidic moieties (Figures S1–S7). The aglycone moiety showed signals for six methyls, nine methylenes, two oxygenated methines, three methines, one olefinic, and nine quaternary carbons. The ^1^H-^1^H COSY couplings of H_2_-1/H-2, H-2/H-3, H_2_-6/H_2_-7, H_2_-15/H_2_-16, H-18/H_2_-19, and H_2_-21/H_2_-22 showed direct connections between the carbons in the aglycone. The notable HMBC correlations of H_3_-24 with C-3 and C-23; H_3_-25 with C-1, C-5, and C-10; H_3_-26 with C-7 and C-8; and H_3_-27 with C-8, C-14, and C-15 indicated the locations of the methyl groups. The correlations of H_3_-24 with C-23, H-18 with C-12 and C-28, and H_2_-22 with C-28 were also key signals indicating the locations of the carbonyl and olefinic groups. The ROE showed H-3/H-5/H-9/H_3_-27correlations. Additionally, H-3/H-1_axial_/H-2 correlations were presented, indicating that H-2, H-3, H-5, H-9, and H_3_-27 are located in the same directions. In addition, H_3_-24/H_3_-25/H_3_-26/H-18, and H-18/H-1_equitorial_ were in the same orientation as H-18, H_3_-24, H_3_-25, H_3_-26, and H-16.

Based on the presented data, the chemical structure of the aglycone in **1** (**1a**) was proposed as a pentacyclic triterpenoid glycoside, 2β,3β-dihydroxyolean-12-ene-23,28-dioic acid, known as medicagenic acid [20,21,22]. Furthermore, **1a** was verified through liquid chromatography quadrupole time-of-flight mass spectrometry (LC-QTOF-MS) and NMR after eliminating the sugar moiety via acid hydrolysis (Figures S31–S34 and S38).

The glycosidic part of pannoside F (**1**) was identified through the presence of anomeric proton signals in the ^1^H NMR spectrum at δ_H_ 4.42 (d, *J* = 7.5 Hz, H-1_gluA_), 5.40 (d, *J* = 8.0 Hz, H-1_rha1_), 5.33 (d, *J* = 2.0 Hz, H-1_rha2_), and 4.53 (d, *J* = 7.5 Hz, H-1_xyl_). The corresponding carbon signals were observed at δ_C_ 104.5 (C-1_gluA_), 94.9 (C-1_rha1_), 101.4 (C-1_rha2_), and 106.9 (C-1_xyl_); these signals aligned with the presence of one *β*-d-glucuronic acid, *β*-d-rhamnose (rha1), acetylated *β*-d-xylose, and one α-l-rhamnose (rha2). The β-configurations of glucuronic acid, xylose, and rhamnose (rha1) were inferred from the large coupling constant (*J* = 7.5–8.0 Hz) of the anomeric proton, whereas those of α-l-rhamnose (rha2) were confirmed by their respective coupling constants (*J* = 2.0 Hz) and chemical shifts [23]. In particular, the downfield shifts observed for C-6 of the rhamnose at δ_C_ 16.8 (rha1) and δ_C_ 18.5 (rha2) indicated esterification at these positions. In addition, the HMBC correlations from H-3_xyl_ (δ_H_ 4.85) of xylose to C-1′_xyl_ (δ_C_ 173.1) and from H-2′_xyl_ (δ_H_ 2.13) to C-1′_xyl_ (δ_C_ 173.1) indicate the presence of acetylated xylose. Additionally, HMBC correlations are crucial in determining the linkages between sugar units and the aglycone.

Significant HMBC correlations were found between H-1_gluA_ (δ_H_ 4.42) and C-3 (δ_C_ 86.9) of the aglycone, indicating that the glucuronic acid was attached at the C-3 position. The HMBC correlations from H-1_rha1_ (δ_H_ 5.40) to C-28 (δ_C_ 178.0) and H-1_rha2_ (δ_H_ 5.33) to C-2_rha1_ (δ_C_ 75.3) confirmed the rhamnose units’ connection at C-28 of the aglycone. Furthermore, H-1_xyl_ (δ_H_ 4.53) to C-4_rha2_ (δ_C_ 84.9) revealed the linkage of three sugar units. The presence of 3-HB units was confirmed by proton signals at δ_H_ 2.71 (dd, *J* = 12.0, 6.5 Hz, H-2′ of 3-HB) and carbon signals at δ_C_ 41.2 (C-2′ of 3-HB). The HMBC correlations between these protons and the carbons at δ_C_ 172.4 (C-1′ of 3-HB) and δ_C_ 68.7 (C-3″ of 3-HB) confirmed the ester linkage of the 3-HB moieties to the rhamnose units. The signals at δ_H_ 5.25 (s, H-3′) and δ_H_ 1.29 (s, H-4′) further established the structure of the 3-HB units on rhamnose (rha1).

**Figure 1:**
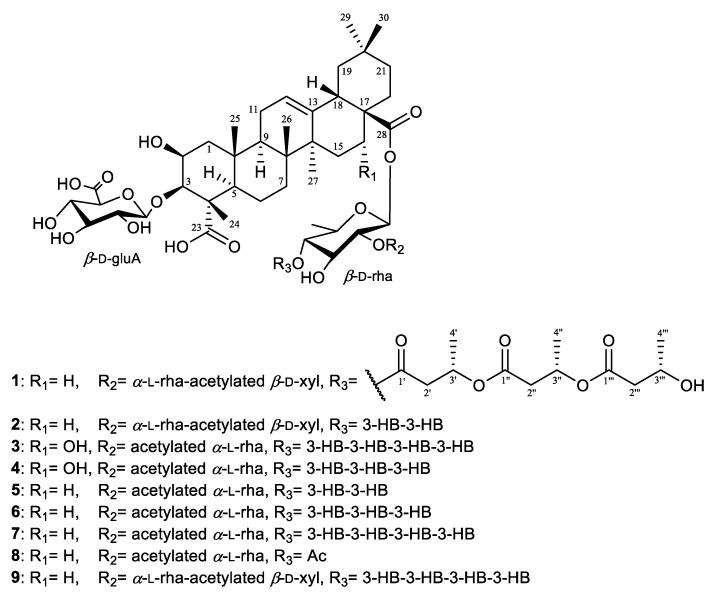
Chemical structures of pannosides F–I (**1**–**4**) and A–E (**5**–**9**).

**Figure 2:**
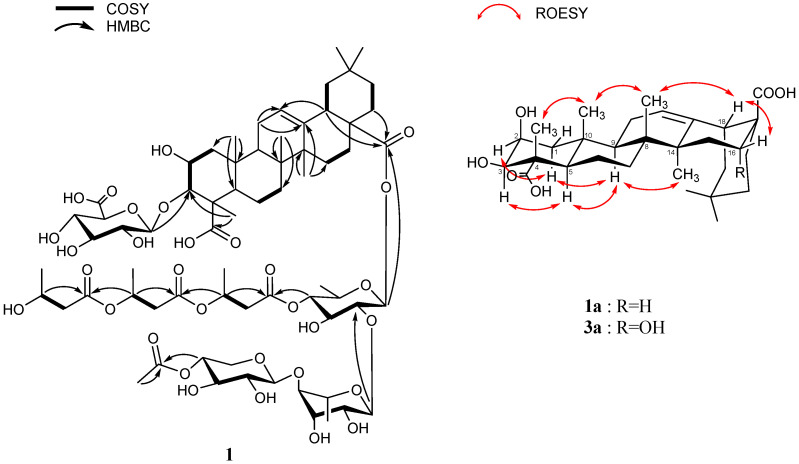
Key COSY, HMBC correlation of **1**, and ROESY correlation of an aglycone.

**Figure 3:**
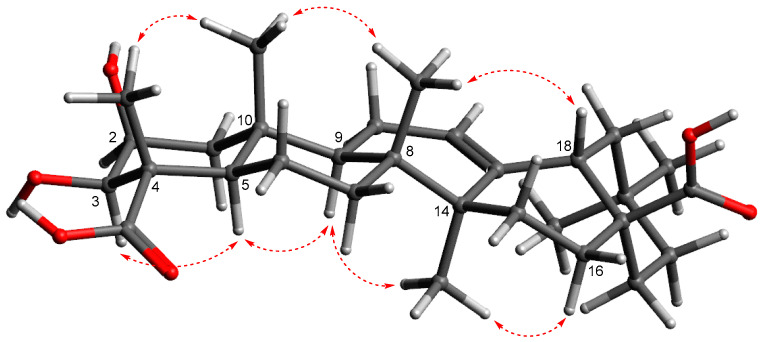
The energy-minimized structure and key ROESY correlations of **1a**.

The hydrolysate of pannoside F (**1**) was prepared by performing acid hydrolysis with 6 N HCl to identify the absolute configurations of the 3-HB residues in **1**. The hydrolysate and authentic standards of *S*- and *R*-3-HB were treated with *S*-phenylglycine methyl ester (*S*-PGME), and the retention times of the *S*-PGME derivatives were compared using LC-QTOF-MS. By comparing the retention time of each *S*-PGME derivative, it was observed that the *S*-PGME derivative of the hydrolysate eluted at the same *R*t (16.5 min) as the *S*-3-HB-*S*-PGME (16.5 min), while differing from the *R*t of *R*-3-HB-*S*-PGME (15.7 min). This result confirms that the absolute configuration of the 3-HB residues was determined to be the *S*-configuration (Figure S39) [24]. On the basis of the comprehensive data from the 1D, 2D NMR, and PGME derivative data, the structure of pannoside F (**1**) was confirmed to be 3-*O*-[*β-*d-glucuronic acid]-28-*O*-[*β*-d-rhamnosyl-4-*O*-*tri*-(*S*)-3-hydroxybutyrate-[*O-α-*l*-*rhamnosyl-(1→2)-*O-β-*d-(3-*O*-acetyl)-xylopyranosyl-(1→4)]-medicagenic acid (Figure 1, Figure 2 and Figure 3, Table 1 and Table 2).

Pannoside G (**2**) was purified as a white amorphous powder, and the molecular formula was determined to be *m*/*z* 1339.5925 [M + Na]^+^ via high-resolution electrospray ionization mass spectrometry (HR-ESI-MS). A comparison of its ^1^H and DEPT-135 spectra with those of pannoside F revealed that pannoside G shares the same aglycone, medicagenic acid, as the base structure. The analysis of 2D NMR spectra, including HSQC and HMBC correlations, confirmed the presence of a β-d-glucuronic acid unit attached at the C-3 position of medicagenic acid and β-d-rhamnose, α-l-rhamnose at the C-28 position. In addition, the acetylated β-d-xylose attached to the 4-position of *α*-l-rhamnose further diversified the structure. The presence of a 3-HB unit attached to the 4-position of β-d-rhamnose was confirmed through further HMBC correlations between H-3′ (δ_H_ 5.25) and C-1″ (δ_C_ 172.6) (Figures S8–S14). Compared to pannoside F, pannoside G has one less acyl chain. On the basis of the above data, the structure of pannoside G was confirmed to be 3-*O*-[*β*-d-glucuronic acid]-28-*O*-[*β*-d-rhamnosyl-4-*O*-*bis*-(*S*)-3-hydroxybutyrate]-[*O*-*α*-l-rhamnosyl-(1→2)-*O*-*β*-d-(3-*O*-acetyl)-xylopyranosyl-(1→4)]-medicagenic acid (Figure 1, Table 1 and Table 2).

Pannoside H (**3**) was isolated as a white powder, and the molecular formula was proven to be *m*/*z* 1437.6297 [M + Na]^+^ by HR-ESI-MS. NMR analysis revealed that pannoside H contained similar aglycones as pannosides F and G, with an additional hydroxyl group at the C-16 position; this was confirmed through a comparison of the chemical shifts in the ^1^H and ^13^C NMR spectra (Figures S15–S21).

The signals of the aglycone moiety indicate the presence of six tertiary methyls, nine methylenes, three oxygenated methines, two methines, one olefinic, and nine quaternary carbons. In particular, proton signals at δ_H_ 4.27 (CH-2), 4.08 (CH-3), and 4.42 (CH-16) and carbon signals at δ_c_ 71.3 (C-2), 86.6 (C-3), and 75.1 (C-16) indicate three oxygenated methines. The proton-proton coupling was confirmed by the COSY spectrum, and the proton-carbon correlation confirmed by the HMBC spectrum was similar to that of pannoside F. The relative configuration was determined from the ROESY spectrum. Through-space couplings of H-3/H-5/H-9 and H-9/H_3_-27specified that H-3, H-5, H-9, and H_3_-27 were located in the same directions. Additionally, H_3_-24/H_3_-25/H_3_-26/H-18 and H-18/H-16 revealed that H-16, H-18, H_3_-24, H_3_-25, and H_3_-26 were in the same orientation. As a result, the chemical structure of the aglycone in **3** (**3a**) was proposed as a pentacyclic triterpenoid glycoside, 2β,3β,16α-trihydroxyolean-12-ene-23,28-dioic acid, named zanhic acid [20,21]. In addition, the aglycone was confirmed through LC-QTOF-MS and NMR by removing the sugar moiety through acid hydrolysis (Figures S35–S38).

The glycosides of pannoside H were determined through the presence of anomeric proton signals in the ^1^H NMR spectrum at δ_H_ 4.40 (d, *J* = 7.5 Hz, H-1_gluA_), 5.41 (d, *J* = 8.0 Hz, H-1_rha1_), and 5.32 (d, *J* = 2.0 Hz, H-1_rha2_). The corresponding carbon signals were observed at δ_C_ 105.1 (C-1_gluA_), 95.2 (C-1_rha1_), and 99.3 (C-1_rha2_); these signals aligned with the presence of one *β*-d-glucuronic acid, *β*-d-rhamnose (rha1), and one acetylated α-l-rhamnose (rha2).

Additionally, HMBC correlations from H-2_rha2_ (δ_H_ 5.33) and H-2′_rha2_ (δ_H_ 2.07) of rhamnose to C-1′_rha2_ (δ_C_ 171.8) and from H-3_rha2_ (δ_H_ 4.99) and H-4′_rha2_ (δ_H_ 1.99) to C-3′_rha2_ (δ_C_ 172.3) revealed the presence of acetylated rhamnose (rha2). Significant HMBC correlations included those from H-1_gluA_ (δ_H_ 4.40) to C-3 (δ_C_ 86.6) of the aglycone, and correlations from H-1_rha1_ (δ_H_ 5.41) to C-28 (δ_C_ 178.2) and from H-1_rha2_ (δ_H_ 5.32) to C-2_rha1_ (δ_C_ 76.4) revealed the positions of three sugar units. In addition, the compound contained four consecutive 3-HB units, and each 3-HB unit exhibited characteristic HMBC correlations, such as H-3‴ (δ_H_ 5.25), which correlated with C-1⁗ (δ_C_ 172.6). These four consecutive 3-HB units created a highly elongated and branched sugar chain at the C-28 position of the aglycone, further increasing the structural complexity of pannoside H. Based on the comprehensive data, the structure of pannoside H was confirmed to be 3-*O*-[*β-*d-glucuronic acid]-28-*O*-[*β*-d-rhamnosyl-4-*O*-*tetra*-(*S*)-3-hydroxybutyrate]-[*O-α-*l-(2,3-*O*-diacetyl)-rhamnosyl-(1→2)-zanhic acid (Figure 1, Table 1 and Table 2).

Pannoside I (**4**) was purified as a white powder, and the molecular formula was determined to be *m*/*z* 1351.5925 [M + Na]^+^ by HR-ESI-MS. Similar to pannoside H, pannoside I featured an identical zanhic acid core with a hydroxyl group at the C-16 position. The sugar units attached to the C-3 and C-28 positions were identical to those of pannoside H, whereas pannoside I contained three consecutive 3-HB units; these results were confirmed by the HMBC correlations between H-3‴ (δ_H_ 4.15) and C-1‴ *(*δ_C_ 172.4), and characteristic shifts in the carbonyl groups and methylene units were observed in the DEPT-135 spectrum. Compared with pannoside H, the absence of the fourth 3-HB unit in pannoside I was noted by the lack of additional carbonyl and methine signals in the NMR spectra (Figures S22–S28). These structural variations in the acyl chain distinguished pannoside I from its analogs. On the basis of the presented data, the structure of pannoside I was confirmed to be 3-*O*-[*β-*d-glucuronic acid]-28-*O*-[*β*-d-rhamnosyl-4-*O*-*tri*-(*S*)-3-hydroxybutyrate]-[*O-α-*l-(2,3-*O*-diacetyl)-rhamnosyl-(1→2)-zanhic acid (Figure 1, Table 1).

**Figure 4:**
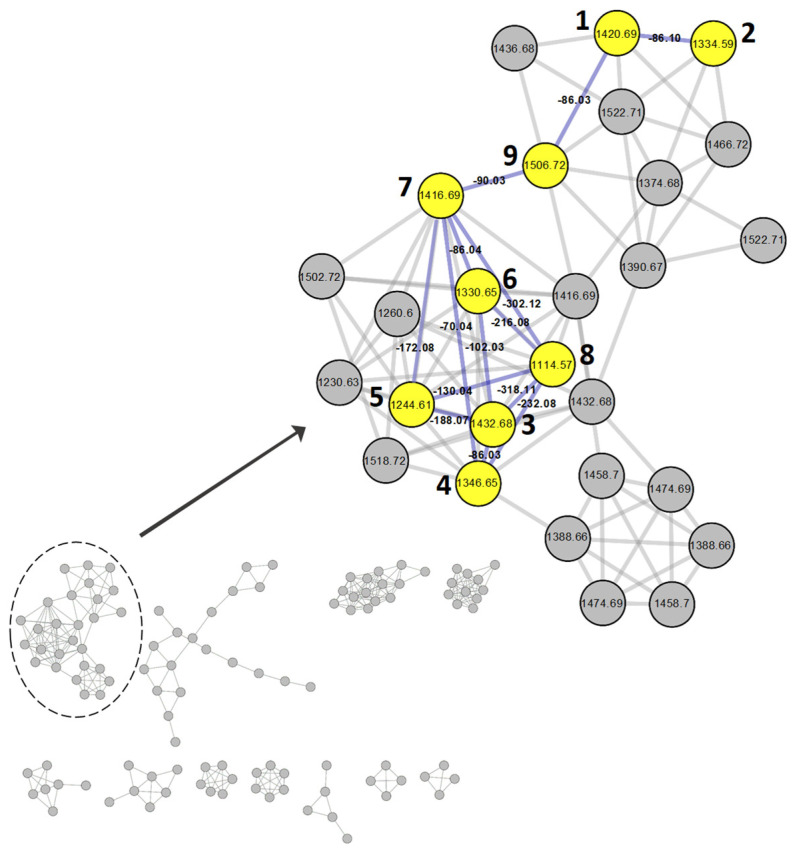
GNPS molecular networking cluster designated as pannoside analogs from the extract of *Aster tripolium*. The numbers beside nodes denote compounds **1**–**9**. **1**, pannoside F (*m*/*z* 1420.69 [M + NH_4_]^+^); **2**, pannoside G (*m*/*z* 1334.59 [M + NH_4_]^+^); **3**, pannoside H (*m*/*z* 1432.68 [M + NH_4_]^+^); **4**, pannoside I (*m*/*z* 1346.65 [M + NH_4_]^+^); **5**, pannoside A (*m*/*z* 1244.61 [M + NH_4_]^+^); **6**, pannoside B (*m*/*z* 1330.65 [M + NH_4_]^+^); **7**, pannoside C (*m*/*z* 1416.69 [M + NH_4_]^+^); **8**, pannoside D (*m*/*z* 1114.57 [M + NH_4_]^+^); **9**, pannoside E (*m*/*z* 1506.72 [M + NH_4_]^+^). MS differences are indicated on the lines between nodes.

Molecular networking revealed that triterpenoid saponins of *A. tripolium* aggregated into a large cluster containing pannosides A–I (**1**–**9**). These results suggest that the MS/MS patterns and chemical structures of these compounds are similar. In addition to the nine nodes, there are twelve more nodes (excluding duplicates) in the cluster, which means that similar triterpenoid saponins are present, but as they were not obtained in small quantities, a bioactivity assay of the nine compounds was conducted (Figure 4).

To evaluate the antiviral activity of pannosides **1**–**9** against EV71, CVB3, and HRV1B, a cell viability assay was conducted using EV71- and CVB3-infected Vero cells and HRV1B-infected HeLa cells. Pannosides **1**–**9** were diluted in 5-fold serial dilutions, with concentrations ranging from 100 µM to 0.8 µM, to evaluate their antiviral activity. Vero cells were infected with EV71 and CVB3, while HeLa cells were infected with HRV1B. Simultaneously, pannosides **1**–**9** were administered at predetermined concentrations. The virus was allowed to infect the cells until the cell viability of the vehicle (virus-infected cells) reached 20%. Afterward, the cells were washed, fixed, and stained with sulforhodamine B (SRB). The majority of pannosides **1**–**9** inhibited the viruses in a concentration-dependent manner. Notably, some extracts exhibited strong antiviral activity at the highest concentration of 100 µM, resulting in high cell viability without toxicity. In particular, the extracts demonstrated greater antiviral activity for HRV1B than for EV71 and CVB3 at the same concentration.

**Figure 5:**
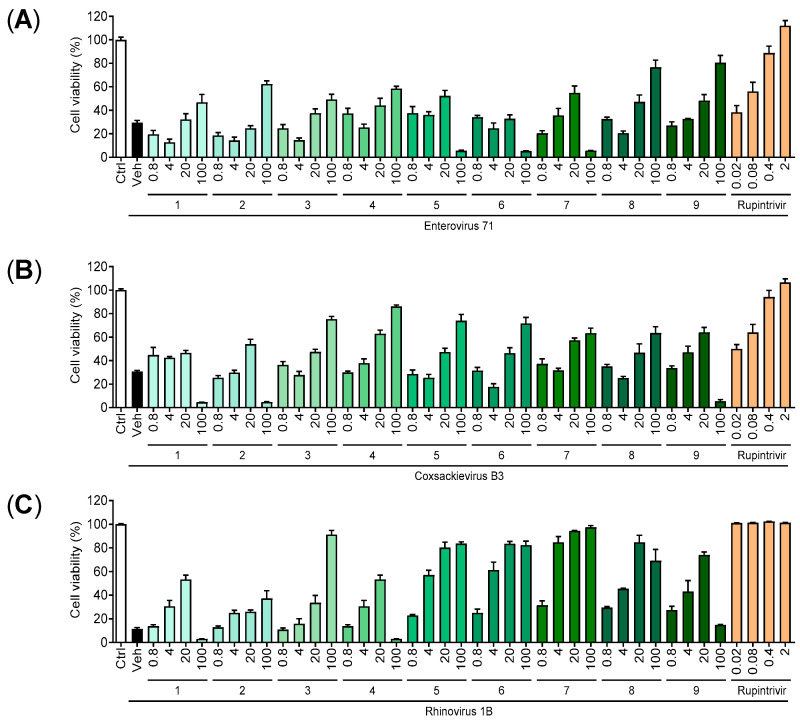
Antiviral activities of pannosides (**1**–**9**) compared to rupintrivir (positive control). Pannosides and rupintrivir were treated to virus added mammalian cells 0.8 to 100 µM and 0.02 to 2 µM, respectively. Antiviral activity against (**A**) enterovirus A71 (EV71) in Vero cells; (**B**) coxsckievirus B3 (CVB3) in Vero cells; (**C**) rhinovirus 1B (HRV1B) in HeLa cells.

In this study, we isolated and characterized the structures of four unreported saponins, pannosides F–I (**1**–**4**), from the halophyte *A*. *tripolium*. These compounds, together with previously identified pannosides A–E (**5**–**9**), were tested for their antiviral activity against enterovirus A71 (EV71), coxsackievirus B3 (CVB3), and rhinovirus 1B (HRV1B). Pannosides exhibited limited activity against EV71 and CVB3; however, pannosides demonstrated notable antiviral effects against HRV1B. Specifically, pannosides A–E (**5**–**9**) presented IC_50_ values ranging from 2.26 to 9.58 µM. In contrast, pannosides F, H, and I (**1**, **3**, and **4**) did not exhibit significant inhibitory effects on HRV1B, and pannoside G (**2**) demonstrated moderate activity (IC_50_ = 50.1 ± 10.8 µM) (Figure 5). While Pannosides A–I share similar structural frameworks, variations in aglycone hydrophobicity, 3-HB chain length, and sugar moiety composition suggest that these factors collectively influence antiviral efficacy [25,26].

## 3. Experimental Section

### 3.1. General Experimental Procedures

Optical rotations were determined using an Optronic P3000 polarimeter (KR*Ü*SS GmbH, Hamburg, Germany). UV spectra were collected via a Cary 100 UV-VIS spectrophotometer (Varian, Santa Clara, CA, USA) with a 1-cm micro quartz cuvette, and IR spectra were obtained via a Cary 630 FTIR (Agilent Technologies, Santa Clara, CA, USA). NMR spectra were obtained via a JEOL 600 MHz instrument (JEOL, Tokyo, Japan) and recorded with CD_3_OD-*d*_4_ solvent. Proton and carbon NMR spectra were measured at 600 MHz and 150 MHz, respectively. An Agilent 6530 iFunnel quadrupole time-of-flight mass spectrometer (Q-TOF-MS) coupled with an Agilent 1290 UHPLC system at 25 °C was used to acquire high-resolution electrospray ionization mass (HR-ESI-MS) spectrometric data. The compounds were purified via an Agilent 1100 series capillary LC system combined with a Waters micromass ZQ mass spectrometer (Waters Corp., Milford, MA, USA).

### 3.2. Plant Collection, Extraction, and Compound Purification

For a halophyte, the bulk of *A*. *tripolium* was collected from a tidal mudflat marsh in Songdo, Incheon, Republic of Korea, in September 2022. The whole parts of *A*. *tripolium* were dried with a dehydrator under 60 °C of hot air for 12 h. The dried sample was ground into powder and extracted twice with 12 L of methanol via a sonicator (40 °C, 3 h). The concentrated crude extract (12.8 g) was loaded onto packed Sephadex LH-20 resin identically to the previous method [12]. Methanol was used as the mobile phase, which was collected at 15-min intervals and divided into a total of 8 fractions (fractions 1–8). Fraction **2** (15–30 min) was further purified via a semi-preparative LC-MS instrument with elution of isocratic 49% aqueous acetonitrile with 0.1% formic acid using a J’sphere ODS-H80 column (20 × 250 mm, 4 mm, Waters Co.), yielding compounds **1** (*t*_R_ = 19.9 min), **2** (*t*_R_ = 16.6 min), **3** (*t*_R_ = 26.9 min), and **4** (*t*_R_ = 21.1 min). The four targeted compounds were **1** (3.4 mg), **2** (2.1 mg), **3** (3.1 mg), and **4** (2.8 mg), which were then specified as pannosides F (**1**), G (**2**), H (**3**), and I (**4**), respectively.

*Pannoside F* (**1**)

White amorphous powder; [*α*]_D_^25^ = +3.6 (*c* 0.1, MeOH); IR *v*_max_ (ATR) 3307, 3258, 1653, 1001 cm^−1^; UV (MeOH): λ_max_ 190, 220 nm; HR-ESI-MS *m*/*z* 1425.6296 [M + Na]^+^ (calcd. for C_67_H_102_O_31_Na *m*/*z* 1425.6297) (Figure S29); ^1^H NMR (CD_3_OD-*d*_4_, 600 MHz); and DEPT-135 (CD_3_OD-*d*_4_, 150 MHz).

*Pannoside G* (**2**)

White amorphous powder; [*α*]_D_^25^ = +2.0 (*c* 0.1, MeOH); IR *v*_max_ (ATR) 3309, 3258, 1652, 1001 cm^−1^; UV (MeOH): λ_max_ 190, 220 nm; HR-ESI-MS *m*/*z* 1339.5925 [M + Na]^+^ (calcd. for C_63_H_96_O_29_Na *m*/*z* 1339.5929) (Figure S29); ^1^H NMR (CD_3_OD-*d*_4_, 600 MHz); and DEPT-135 (CD_3_OD-*d*_4_, 150 MHz).

*Pannoside H* (**3**)

White amorphous powder; [*α*]_D_^25^ = +3.2 (*c* 0.1, MeOH); IR *v*_max_ (ATR) 3307, 3309, 1608, 1110, 1001 cm^−1^; UV (MeOH): λ_max_ 190, 223 nm; HR-ESI-MS *m*/*z* 1437.6297 [M + Na]^+^ (calcd. for C_68_H_102_O_31_Na *m*/*z* 1437.6297) (Figure S29); ^1^H NMR (CD_3_OD-*d*_4_, 600 MHz); and ^13^C NMR (CD_3_OD-*d*_4_, 150 MHz).

*Pannoside I* (**4**)

White amorphous powder; [*α*]_D_^25^ = +2.6 (*c* 0.1, MeOH); IR *v*_max_ (ATR) 3315, 3298, 1609, 1110, 1001 cm^−1^; UV (MeOH): λ_max_ 190, 223 nm; HR-ESI-MS *m*/*z* 1351.5925 [M + Na]^+^ (calcd. for C_64_H_96_O_29_Na *m*/*z* 1351.5929) (Figure S29); ^1^H NMR (CD_3_OD-*d*_4_, 600 MHz); and DEPT-135 (CD_3_OD-*d*_4_, 150 MHz).

*Medicagenic acid* (**1a**)

White amorphous powder; [*α*]_D_^25^ = +26 (*c* 1.0, MeOH); IR *v*_max_ (ATR) 3398, 2996, 1610 cm^−1^; UV (MeOH): λ_max_ 197 nm; HR-ESI-MS *m*/*z* 501.3227 [M − H]^−^ (calcd. for C_30_H_45_O_6_ *m*/*z* 501.3221) (Figure S38).

*Zanhic acid* (**3a**)

White amorphous powder; [*α*]_D_^25^ = +20 (*c* 0.8, MeOH); IR *v*_max_ (ATR) 3485, 2950, 1601, 1353 cm^−1^; UV (MeOH): λ_max_ 198 nm; HR-ESI-MS *m*/*z* 517.3154 [M − H]^−^ (calcd. for C_30_H_45_O_7_ *m*/*z* 517.3170) (Figure S38).

### 3.3. Determination of the Absolute Configuration of 3-HB Residues in Pannosides

Pannoside F (**1**, 1 mg) was acid hydrolyzed by 6 N HCl at 115 °C for 2 h to obtain 3-HB residues, after which the reactant was rapidly chilled by soaking the reaction vial in ice water. The reaction solvent was evaporated with a vacuum evaporator, and any remaining HCl was removed overnight via a lyophilizer. The 3-HB residues in the dried hydrolysate and authentic standards of *S-* and *R*-3-HB were dissolved in 2 mL of tetrahydrofuran. Then, the samples were treated with 10 mg of *S*-PGME, 10 mg of 1-(3-dimethylaminopropyl)-3-ethylcarbodiimide hydrochloride (EDC HCl), and 10 mg of 4-dimethylaminopyridine (4-DMAP). The reactants were analyzed using LC-QTOF-MS through the following method, after the reaction mixtures were stirred at room temperature for 1 h: 10–50% aqueous acetonitrile with 0.1% formic acid for 40 min and a YMC-Triart C18 column (150 × 2.0 mm, 5 μm).

### 3.4. Analyzes of Metabolites and Molecular Networking

To analyze the metabolites of *A*. *tripolium*, molecular networks based on tandem mass spectrometry were constructed via the Global Natural Product Social Molecular Network (GNPS) platform [27]. The dried extract of *A*. *tripolium* was dissolved in methanol as a concentration of 250 μg/mL and analyzed with LC-MS using a YMC-Triart C18 column (150 × 2.0 mm, 5 μm) (YMC Korea, Republic of Korea). The following conditions were used to conduct the MS experiment: a drying gas temperature of 300 °C, a drying gas flow rate of 8 L/min, a sheath gas temperature of 350 °C, a sheath gas flow rate of 11 L/min, a capillary voltage of +3.5 kV, and use of positive mode. The MS/MS data of the *A*. *tripolium* extract were converted to a GNPS-compatible format, mzML, with the MS-Convert program, and the converted files were utilized to build an MS/MS molecular network via the GNPS web server. The parameters were set as follows: precursor ion mass tolerance, 2.0 Da; product ion tolerance, 0.05 Da; molecular network cosine score, 0.5; minimum number of matched fragment ions, 6; and minimum cluster size, 2. After analysis, the data were visualized with Cytoscape 3.10.1 software [28].

### 3.5. Biological Assays

#### 3.5.1. Cell Culture and Viruses

Enterovirus A71 and coxsackievirus B3 (obtained from the American Type Culture Collection, USA) were propagated at 37 °C in Vero cells, and rhinovirus 1B (ATCC) was propagated at 33 °C in HeLa cells. In a 37 °C incubator with 5% CO_2_ (SANYOElectric Co., Osaka, Japan), Vero cells and HeLa cells (ATCC) were cultured in Dulbecco’s modified Eagle’s medium (DMEM) supplemented with 10% heat-inactivated fetal bovine serum and 0.01% antibiotic–antimycotic solution. DMEM, fetal bovine serum, trypsin–EDTA, and antibiotic–antimycotic solution were procured from Corning (Corning Incorporated, Corning, NY, USA). Falcon (BD Biosciences, Franklin Lakes, NJ, USA) was the purchase source for the tissue culture plates.

#### 3.5.2. Antiviral Activity Assay

The SRB assay was employed to assess cytotoxicity and antiviral activity through cytopathic effects (CPEs) [29]. A 96-well culture plate was seeded with 3 × 10^4^ cells per well one day prior to infection. The cells were washed with 1× phosphate-buffered saline (PBS) (Corning Incorporated, USA) the following day, after the culture medium was aspirated. The SRB method was employed to monitor CPE by determining the infectivity of each virus [30]. This method allows for the determination of the percentage of viable cells. The diluted virus suspensions of EV71, CVB3, and HRV1B were added to mammalian cells in a volume of 0.09 mL, as determined by the viability of mammalian cells for each virus. This dosage was chosen to generate suitable CPEs 48 h following infection. The antiviral activity of each test compound was assessed through fivefold dilutions, which ranged from 0.8 to 100 µM. We utilized three wells for the viral control (virus-infected and nondrug-treated cells) and the cell control (noninfected and nondrug-treated cells). Sigma-Aldrich (Burlington, MA, USA) was the source of the Rupintrivir and SRB. The cells were incubated in 96-well culture plates at 37 °C with 5% CO_2_ for 2–3 days for EV71 and CVB3, and at 33 °C with 5% CO_2_ for HRV1B until 70–80% CPE was observed. The wells were thoroughly washed twice with PBS, and the supernatant was discarded at the conclusion of the culture. The cells were fixed with ice-cold 70% acetone (100 µL/well) and subsequently stained with 0.4% SRB in 1% acetic acid. The SpectraMax^®^ i3 microplate reader (Molecular Devices, USA) was used to measure the absorbance at 562 nm, with a reference absorbance of 620 nm. The Core-Facility for Innovative Cancer Drug Discovery (CFICDD) at Kangwon National University was used to measure the IR spectra. The results were subsequently converted to percentages of the controls. Additionally, the following formula was used to calculate the percentage of protection achieved by the test compound in the virus-infected cells: (ODt)virus − (ODc)virus) ÷ ((ODc)mock − (ODc)virus) × 100%, where (ODt)virus is the optical density measured in virus-infected cells with a specific concentration of the test compound, (ODc)virus is the optical density of the nondrug-treated, virus-infected control cells, and (ODc)mock isw the optical density of the non-infected cells [31].

## Figures and Tables

**Table 1:** ^1^H and ^13^C NMR spectroscopic data of aglycone of pannosides F–I (**1**–**4**).

		1			2					3			4		
Position		*δ*_H_	Mult	*δ*_C_	*δ*_H_	Mult	*δ*_C_	Position		*δ*_H_	Mult	*δ*_C_	*δ*_H_	Mult	*δ*_C_
			(*J* in Hz)			(*J* in Hz)					(*J* in Hz)			(*J* in Hz)	
1	CH_2_	1.27	m	44.8	1.27	m	44.9	1	CH_2_	1.27	d (6.5)	44.9	1.28	m	44.8
		2.13	m		2.13	m				2.12	m		2.11	m	
2	CH	4.29	m	71.3	4.27	t (3.5)	71.2	2	CH	4.27	m	71.3	4.35	m	71.0
3	CH	4.07	m	86.9	4.07	m	86.7	3	CH	4.08	m	86.6	4.08	m	86.5
4	C			53.3			53.3	4	C			53.4			53.3
5	CH	1.60	m	53.2	1.61	m	53.3	5	CH	1.57	m	53.4	1.60	m	53.1
6	CH_2_	1.18	m	21.7	1.18	m	21.8	6	CH_2_	1.18	m	21.7	1.12	m	21.5
		1.67	m		1.67	m				1.63	m		1.64	m	
7	CH_2_	1.38	m	33.5	1.38	m	33.4	7	CH_2_	1.37 *	m	33.7	1.34	m	33.4
		1.51	m		1.51	m							1.48	m	
8	C			41.0			41.1	8	C			41.3			41.3
9	CH	1.60	m	49.5	1.60	m	49.3	9	CH	1.60	m	49.7	1.60	m	49.5
10	C			37.4			37.4	10	C			37.5			37.3
11	CH_2_	1.95	m	24.8	1.95	m	24.9	11	CH_2_	1.95	m	24.8	1.95	m	24.6
		2.02			2.01	m				2.01	m		2.01	m	
12	CH	5.28	m	123.5	5.29	m	123.5	12	CH	5.27	m	123.7	5.27	m	123.7
13	C			144.5			144.8	13	C			145.0			145.2
14	C			43.3			43.3	14	C			43.3			43.1
15	CH_2_	1.18	m	29.0	1.18	m	28.9	15	CH_2l_	1.41	m	36.5	1.42 *	m	36.5
		1.60	m		1.59	m				1.70	m				
16	CH_2_	1.63	m	23.9	1.64	m	24.2	16	CH	4.42	m	75.1	4.43	m	75.1
		2.07	m		2.06	m									
17	C			48.1			48.1	17	C			48.4			48.1
18	CH	2.80	m	42.9	2.81	m	43.1	18	CH	2.82	m	43.0	2.83	m	42.9
19	CH_2_	1.16	m	47.3	1.16	m	47.3	19	CH_2_	1.15	m	47.6	1.14	m	47.5
		1.74	m		1.73	m				1.73	m		1.73	m	
20	C			31.4			31.6	20	C			31.6			31.4
21	CH_2_	1.25	m	34.9	1.24	m	34.9	21	CH_2_	1.24	m	35.1	1.23	m	34.9
		1.41	m		1.41	m				1.42	m		1.43	m	
22	CH_2_	1.60	m	33.0	1.61	m	33.0	22	CH_2_	1.60	m	33.3	1.61 *	m	33.3
		1.74	m		1.74	m				1.76	m				
23	C			181.6			181.7	23	C			181.8			181.8
24	CH_3_	1.39	s	13.8	1.41	s	13.7	24	CH_3_	1.39	s	13.8	1.39	s	13.7
25	CH_3_	1.29	s	17.5	1.29	s	17.5	25	CH_3_	1.30	s	17.4	1.30	s	17.5
26	CH_3_	0.81	s	17.9	0.80	s	18.0	26	CH_3_	0.83	s	18.0	0.83	s	17.9
27	CH_3_	1.18	s	26.3	1.18	s	26.3	27	CH_3_	1.15	s	26.3	1.15	s	26.4
28	C			178.0			178.1	28	C			178.2			n.d.
29	CH_3_	0.94	s	24.1	0.95	s	24.3	29	CH_3_	0.94	s	24.3	0.94	s	24.2
30	CH_3_	0.91	s	33.4	0.91	s	33.7	30	CH_3_	0.90	s	33.6	0.91	s	33.5

Measured in CD_3_OD-*d*_4_, 150 MHz (^13^C NMR), 600 MHz (^1^H NMR) * overlapped signal.

**Table 2:** ^1^H and ^13^C NMR spectroscopic data of the sugar and 3-HB moieties of pannosides F–I (**1**–**4**).

		1			2					3			4		
Position		*δ*_H_	Mult (*J* in Hz)	*δ*_C_	*δ*_H_	Mult (*J* in Hz)	*δ*_C_	Position		*δ*_H_	Mult (*J* in Hz)	*δ*_C_	*δ*_H_	Mult (*J* in Hz)	*δ*_C_
gluA								gluA							
1	CH	4.42	d (7.5)	104.8	4.41	d (7.5)	104.8	1	CH	4.40	d (7.5)	105.1	4.36	d (7.5)	105.1
2	CH	3.26	m	74.7	3.27	m	74.9	2	CH	3.25	m	74.9	3.25	m	75.0
3	CH	3.37	m	77.2	3.37	m	77.2	3	CH	3.35	dd (9.0, 2.5)	77.3	3.37	m	77.6
4	CH	3.79	m	76.3	3.79	m	76.5	4	CH	3.76	m	76.2	3.77	m	76.3
5	CH	3.49	m	73.1	3.49	m	73.4	5	CH	3.50	m	73.3	3.44	m	73.5
6	C			172.5			172.7	6	C			172.8			172.6
rha-1								rha-1							
1	CH	5.40	d (8.0)	94.9	5.40	d (8.0)	94.9	1	CH	5.41	d (8.0)	95.2	5.42	d (8.0)	95.1
2	CH	3.76	m	75.3	3.80	m	74.7	2	CH	3.80	m	76.4	3.75	dd (9.5, 8.0)	76.2
3	CH	3.88	m	74.8	3.90	m	74.9	3	CH	3.96	m	74.3	3.95	dd (9.5, 3.5)	74.3
4	CH	5.10	m	75.5	5.09	m	75.5	4	CH	5.10	d (3.5)	75.5	5.10	dd (3.5, 1.0)	75.4
5	CH	3.84	m	71.1	3.84	m	71.1	5	CH	3.86	m	71.1	3.85	m	71.0
6	CH_3_	1.07	d (6.0)	16.8	1.08	m	16.7	6	CH_3_	1.07	d (6.5)	16.6	1.07	d (6.5)	16.6
rha-2								rha-2							
1	CH	5.33	d (2.0)	101.4	5.34	d (2.0)	101.5	1	CH	5.32	d (2.0)	99.3	5.32	d (2.0)	99.2
2	CH	3.93	dd (3.5, 2.0)	72.0	3.93	dd (3.5, 2.0)	72.0	2	CH	5.33	m	71.5	5.32	m	71.4
3	CH	3.81	m	72.6	3.82	m	72.4	3	CH	4.99	dd (10.0, 3.5)	73.5	5.00	dd (10.0, 3.5)	73.2
4	CH	3.51	m	84.9	3.54	m	85.2	4	CH	3.49	m	71.1	3.50	t (10.0)	71.1
5	CH	3.80	m	68.8	3.81	m	68.9	5	CH	3.88	m	70.6	3.89	dd (10.0, 6.0)	70.7
6	CH_3_	1.30	d (6.5)	18.5	1.30	m	18.5	6	CH_3_	1.29	m	18.2	1.29	m	18.3
xyl								1′	C			171.8			171.6
1	CH	4.53	d (7.5)	106.9	4.52	d (7.5)	106.8	2′	CH_3_	2.07	s	20.8	2.08	s	20.8
2	CH	3.31	m	74.3	3.31	m	74.4	3′	C			172.3			172.2
3	CH	4.85	m	79.4	4.86	m	79.4	4′	CH_3_	1.99	s	21.0	1.99	s	21.0
4	CH	3.59	m	69.6	3.59	m	69.6								
5	CH_2_	3.27	m	66.9	3.26	m	67.2	3-HB							
		3.87	m		3.87	m		1′	C			171.9			171.9
1′	C			173.1			172.9	2′	CH_2_	2.72 *	dd (16.0, 6.0)	41.5	2.71 *	dd (16.0, 6.0)	41.5
2′	CH_3_	2.13	s	21.3	2.13	s	21.4	3′	CH	5.25	dd (7.5, 6.0)	69.2	5.26	m	69.9
								4′	CH_3_	1.32	m	20.1	1.39	m	13.7
3-HB								1″	C			171.4			171.2
1′	C			172.4			172.0	2″	CH_2_	2.58	m	41.8	2.58 *	m	41.8
2′	CH_2_	2.71	dd (12.0, 6.5)	41.2	2.58 *	m	41.7			2.63					
3′	CH	5.25	m	69.0	5.25	m	68.9	3″	CH	5.25	m	68.9	5.29	m	68.9
4′	CH_3_	1.29	d (6.5)	19.9	1.28	d (6.5)	20.3	4″	CH_3_	1.27	d (6.5)	20.1	1.29	m	17.2
1″	C			171.2			172.6	1‴	C			171.4			172.4
2″	CH_2_	2.55 *	m	41.8	2.41 *	m	45.1	2‴	CH_2_	2.58	m	41.8	2.42 *	m	45.0
										2.63	m				
3″	CH	5.24	m	68.7	4.14	m	65.6	3‴	CH	5.25	m	68.9	4.15	t (6.5)	65.6
4″	CH_3_	1.25	m	19.9	1.20	d (6.5)	23.5	4‴	CH_3_	1.27	m	20.1	1.20	d (6.5)	23.4
1‴	C			172.5				1⁗	C			172.6			
2‴	CH_2_	2.38	dd (6.0, 2.5)	44.9				2⁗	CH_2_	2.41 *	m	45.2			
3‴	CH	4.15	dt (7.5, 6.0)	65.4				3⁗	CH	4.14	q (6.5)	65.6			
4‴	CH_3_	1.18	d (6.0)	23.2				4⁗	CH_3_	1.19	d (6.5)	23.4			

Measured in CD_3_OD -*d*_4_, 150 MHz (^13^C NMR), 600 MHz (^1^H NMR) * overlapped signal.

## Data Availability

Data are contained in the article or Supplementary Materials, further inquiries can be directed to the corresponding author.

## Supplementary Materials

The following supporting information can be downloaded at: https://www.mdpi.com/article/10.3390/md22120524/s1, Figure S1. ^1^H NMR spectrum (600 MHz) of pannoside F (**1**) in CD_3_OD-*d*_4_; Figure S2. DEPT-135 spectrum (150 MHz) of pannoside F (**1**) in CD_3_OD-*d*_4_; Figure S3. COSY NMR spectrum of pannoside F (**1**) in CD_3_OD-*d*_4_; Figure S4. ROESY NMR spectrum of pannoside F (**1**) in CD_3_OD-*d*_4_; Figure S5. TOCSY NMR spectrum of pannoside F (**1**) in CD_3_OD-*d*_4_; Figure S6. HSQC NMR spectrum of pannoside F (**1**) in CD_3_OD-*d*_4_; Figure S7. HMBC NMR spectrum of pannoside F (**1**) in CD_3_OD-*d*_4_; Figure S8. ^1^H NMR spectrum (600 MHz) of pannoside G (**2**) in CD_3_OD-*d*_4_; Figure S9. DEPT-135 spectrum (150 MHz) of pannoside G (**2**) in CD_3_OD-*d*_4_; Figure S10. COSY NMR spectrum of pannoside G (**2**) in CD_3_OD-*d*_4_; Figure S11. ROESY NMR spectrum of pannoside G (2) in CD_3_OD-*d*_4_; Figure S12. TOCSY NMR spectrum of pannoside G (**2**) in CD_3_OD-*d*_4_; Figure S13. HSQC NMR spectrum of pannoside G (**2**) in CD_3_OD-*d*_4_; Figure S14. HMBC NMR spectrum of pannoside G (**2**) in CD_3_OD-*d*_4_; Figure S15. ^1^H NMR spectrum (600 MHz) of pannoside H (**3**) in CD_3_OD-*d*_4_; Figure S16. ^13^C NMR spectrum (150 MHz) of pannoside H (**3**) in CD_3_OD-*d*_4_; Figure S17. COSY NMR spectrum of pannoside H (**3**) in CD_3_OD-*d*_4_; Figure S18. ROESY NMR spectrum of pannoside H (**3**) in CD_3_OD-*d*_4_; Figure S19. TOCSY NMR spectrum of pannoside H (**3**) in CD_3_OD-*d*_4_; Figure S20. HSQC NMR spectrum of pannoside H (**3**) in CD_3_OD-*d*_4_; Figure S21. HMBC NMR spectrum of pannoside H (**3**) in CD_3_OD-*d*_4_; Figure S22. ^1^H NMR spectrum (600 MHz) of pannoside I (**4**) in CD_3_OD-*d*_4_; Figure S23. DEPT-135 spectrum (150 MHz) of pannoside I (**4**) in CD_3_OD-*d*_4_; Figure S24. COSY NMR spectrum of pannoside I (**4**) in CD_3_OD-*d*_4_; Figure S25. ROESY NMR spectrum of pannoside I (**4**) in CD_3_OD-*d*_4_; Figure S26. TOCSY NMR spectrum of pannoside I (**4**) in CD_3_OD-*d*_4_; Figure S27. HSQC NMR spectrum of pannoside I (**4**) in CD_3_OD-*d*_4_; Figure S28. HMBC NMR spectrum of pannoside I (**4**) in CD_3_OD-*d*_4_; Figure S29. Extracted-ion chromatogram (EIC) of pannosides F–I (**1**–**4**); Figure S30. The experimental CD spectra of pannosides F–I (**1**–**4**); Figure S31. ^1^H NMR spectrum (600 MHz) of an aglycone of **1** (**1a**) in CD3OD-*d*4; Figure S32. ^13^C NMR spectrum (150 MHz) of an aglycone of **1** (**1a**) in CD_3_OD-*d*_4_; Figure S33. COSY NMR spectrum (600 MHz) of an aglycone of **1** (**1a**) in CD_3_OD-*d*_4_; Figure S34. ROESY NMR spectrum (600 MHz) of an aglycone of **1** (**1a**) in CD_3_OD-*d*_4_; Figure S35. ^1^H NMR spectrum (400 MHz) of an aglycone of **3** (**3a**) in CD_3_OD-*d*_4_; Figure S36. COSY NMR spectrum (400 MHz) of an aglycone of **3** (**3a**) in CD_3_OD-*d*_4_; Figure S37. ROESY NMR spectrum (400 MHz) of an aglycone of **3** (**3a**) in CD_3_OD-*d*_4_; Figure S38. Extracted-ion chromatograms (EIC) of **1a** and **3a**; Figure S39. EIC of *S*-PGME derivatives; authentic (*S*)-, (*R*)-3-HB and 3-HB residues in hydrolysate of **1**.
