# Supplementary material for: Structural Elucidation and Antiviral Properties of Pannosides from the Halophyte Aster tripolium L
Source: Mar Drugs. 2024 Nov 21;22(12):524. doi: 10.3390/md22120524 (PMC11677766; doi:10.3390/md22120524)

Structure Elucidation and Antiviral Properties of Pannosides from the Halophyte *Aster tripolium* L.

Jaeyoun Lee [1], Jae-Hyoung Song [2], Seo-Hyeon Mun [2], Hyun-Jeong Ko [2], Soohyun Um [1,*], and Seung Hyun Kim [1,*]

1 College of Pharmacy, Yonsei Institute of Pharmaceutical Sciences, Yonsei University, Incheon 21983, South Korea; jaeyoun1024@yonsei.ac.kr

2 Department of Pharmacy, Kangwon National University, Chuncheon 24341, Republic of Korea; thdwohud@naver.com (J.-H.S.); moonnari0606@gmail.com (S.-H.M.); hjko@kangwon.ac.kr (H.-J.K.)

* Correspondence: soohyunum@yonsei.ac.kr (S.U.); kimsh11@yonsei.ac.kr (S.H.K.)

Table of Contents

S3 : **Figure S1.** ^{1}H NMR spectrum (600 MHz) of pannoside F (**1**) in CD_3OD-d_4.
Figure S2. DEPT-135 spectrum (150 MHz) of pannoside F (**1**) in CD_3OD-d_4.
S4 : **Figure S3.** COSY NMR spectrum of pannoside F (**1**) in CD_3OD-d_4.
Figure S4. ROESY NMR spectrum of pannoside F (**1**) in CD_3OD-d_4.
S5 : **Figure S5.** TOCSY NMR spectrum of pannoside F (**1**) in CD_3OD-d_4.
Figure S6. HSQC NMR spectrum of pannoside F (**1**) in CD_3OD-d_4.
S6 : **Figure S7.** HMBC NMR spectrum of pannoside F (**1**) in CD_3OD-d_4.
S7 : **Figure S8.** 1H NMR spectrum (600 MHz) of pannoside G (**2**) in CD_3OD-d_4.
Figure S9. DEPT-135 spectrum (150 MHz) of pannoside G (**2**) in CD_3OD-d_4.
S8 : **Figure S10.** COSY NMR spectrum of pannoside G (**2**) in CD_3OD-d_4.
Figure S11. ROESY NMR spectrum of pannoside G (**2**) in CD_3OD-d_4.
S9 : **Figure S12.** TOCSY NMR spectrum of pannoside G (**2**) in CD_3OD-d_4.
Figure S13. HSQC NMR spectrum of pannoside G (**2**) in CD_3OD-d_4.
S10 : **Figure S14.** HMBC NMR spectrum of pannoside G (**2**) in CD_3OD-d_4.
S11 : **Figure S15.** ^{1}H NMR spectrum (600 MHz) of pannoside H (**3**) in CD_3OD-d_4.
Figure S16. ^{13}C NMR spectrum (150 MHz) of pannoside H (**3**) in CD_3OD-d_4.
S12 : **Figure S17.** COSY NMR spectrum of pannoside H (**3**) in CD_3OD-d_4.
Figure S18. ROESY NMR spectrum of pannoside H (**3**) in CD_3OD-d_4.
S13 : **Figure S19.** TOCSY NMR spectrum of pannoside H (**3**) in CD_3OD-d_4.
Figure S20. HSQC NMR spectrum of pannoside H (**3**) in CD_3OD-d_4.
S14 : **Figure S21.** HMBC NMR spectrum of pannoside H (**3**) in CD_3OD-d_4.
S15 : **Figure S22.** ^{1}H NMR spectrum (600 MHz) of pannoside I (**4**) in CD_3OD-d_4.
Figure S23. DEPT-135 spectrum (150 MHz) of pannoside I (**4**) in CD_3OD-d_4.
S16 : **Figure S24.** COSY NMR spectrum of pannoside I (**4**) in CD_3OD-d_4.
Figure S25. ROESY NMR spectrum of pannoside I (**4**) in CD_3OD-d_4.
S17 : **Figure S26.** TOCSY NMR spectrum of pannoside I (**4**) in CD_3OD-d_4.
Figure S27. HSQC NMR spectrum of pannoside I (**4**) in CD_3OD-d_4.
S18 : **Figure S28.** HMBC NMR spectrum of pannoside I (**4**) in CD_3OD-d_4.
S19 : **Figure S29.** Extracted-ion chromatogram (EIC) of pannosides F–I (**1**–**4**).
Figure S30. The experimental CD spectra of pannosides F–I (**1**–**4**).
S20 : **Figure S31**. ^{1}H NMR spectrum (600 MHz) of an aglycone of **1** (**1a**) in CD_3OD-d_4.
Figure S32. ^{13}C NMR spectrum (150 MHz) of an aglycone of **1** (**1a**) in CD_3OD-d_4.
S21 : **Figure S33**. COSY NMR spectrum (600 MHz) of an aglycone of **1** (**1a**) in CD_3OD-d_4.
Figure S34. ROESY NMR spectrum (600 MHz) of an aglycone of **1** (**1a**) in CD_3OD-d_4.
S22 : **Figure S35**. ^{1}H NMR spectrum (400 MHz) of an aglycone of **3** (**3a**) in CD_3OD-d_4.
Figure S36. COSY NMR spectrum (400 MHz) of an aglycone of **3** (**3a**) in CD_3OD-d_4.
S23 : **Figure S37**. ROESY NMR spectrum (400 MHz) of an aglycone of **3** (**3a**) in CD_3OD-d_4.
S24 : **Figure S38**. Extracted-ion chromatograms (EIC) of **1a** and **3a**.
S25 : **Figure S39.** EIC of *S*-PGME derivatives; authentic (*S*)-, (*R*)-3-HB and 3-HB residues in hydrolysate of **1**.

Figure S1. ^{1}H NMR spectrum (600 MHz) of pannoside F (**1**) in CD_3OD-d_4.

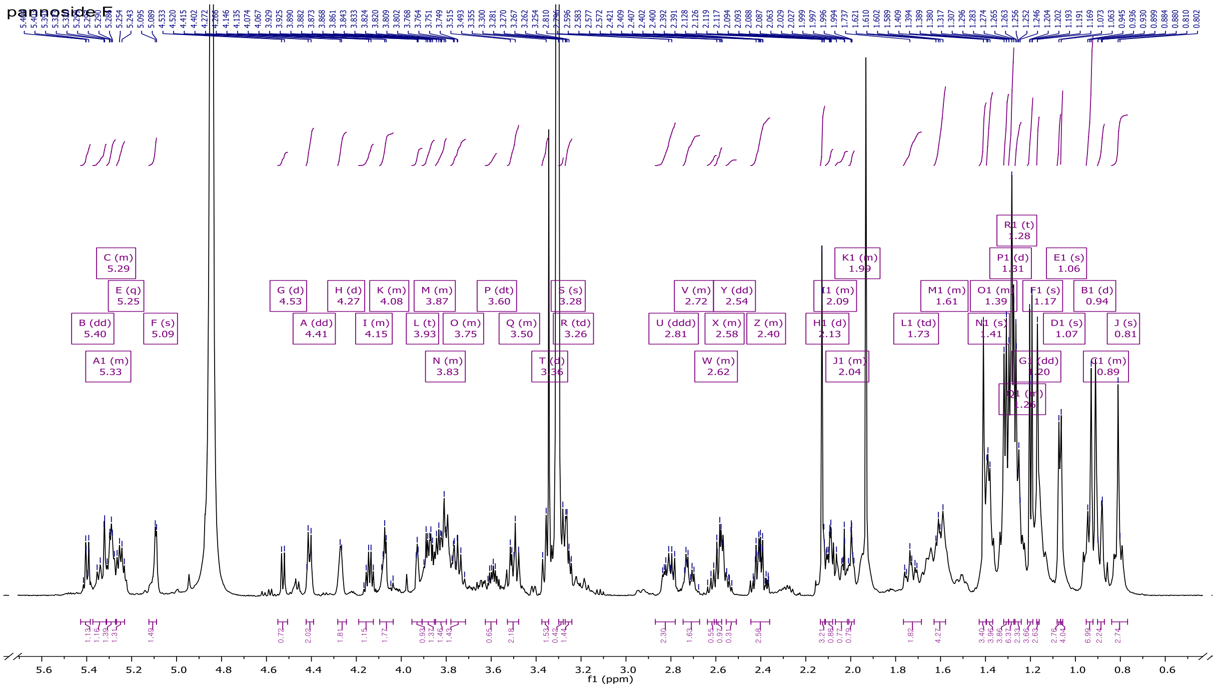

Figure S2. DEPT-135 spectrum (150 MHz) of pannoside F (**1**) in CD_3OD-d_4.

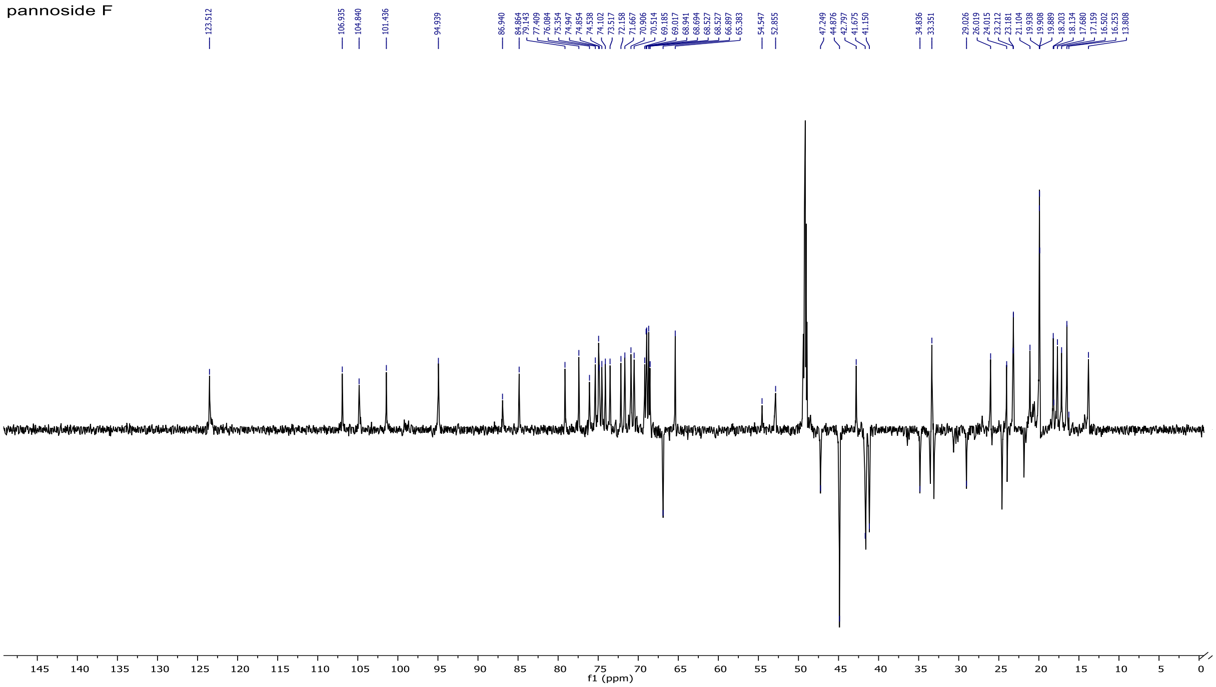

Figure S3. COSY NMR spectrum of pannoside F (**1**) in CD3OD-*d*4.

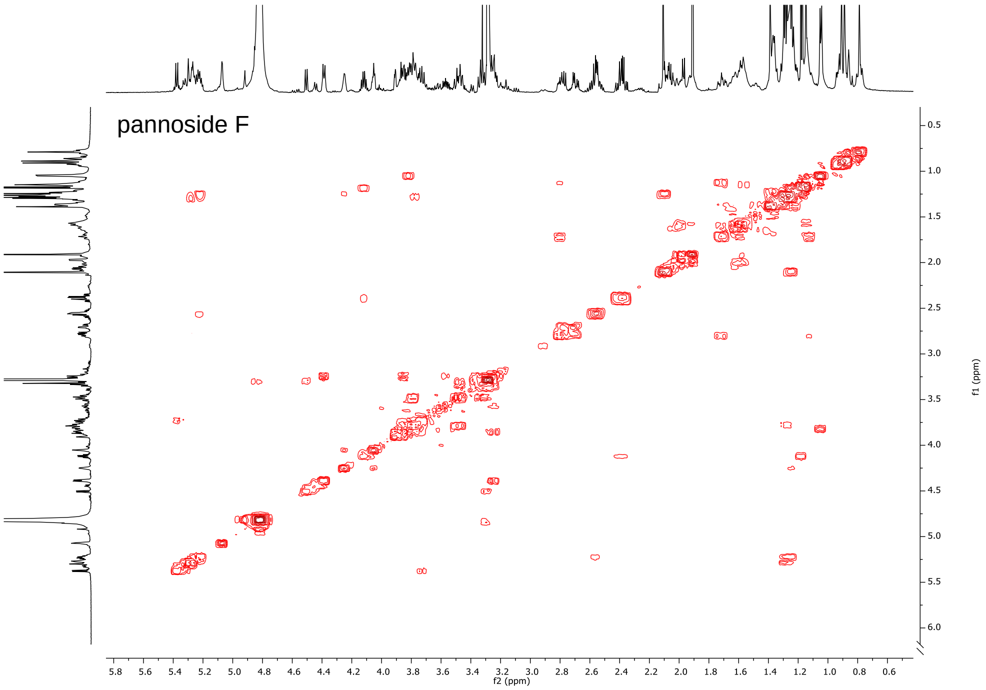

Figure S4. ROESY NMR spectrum of pannoside F (**1**) in CD3OD-*d*4.

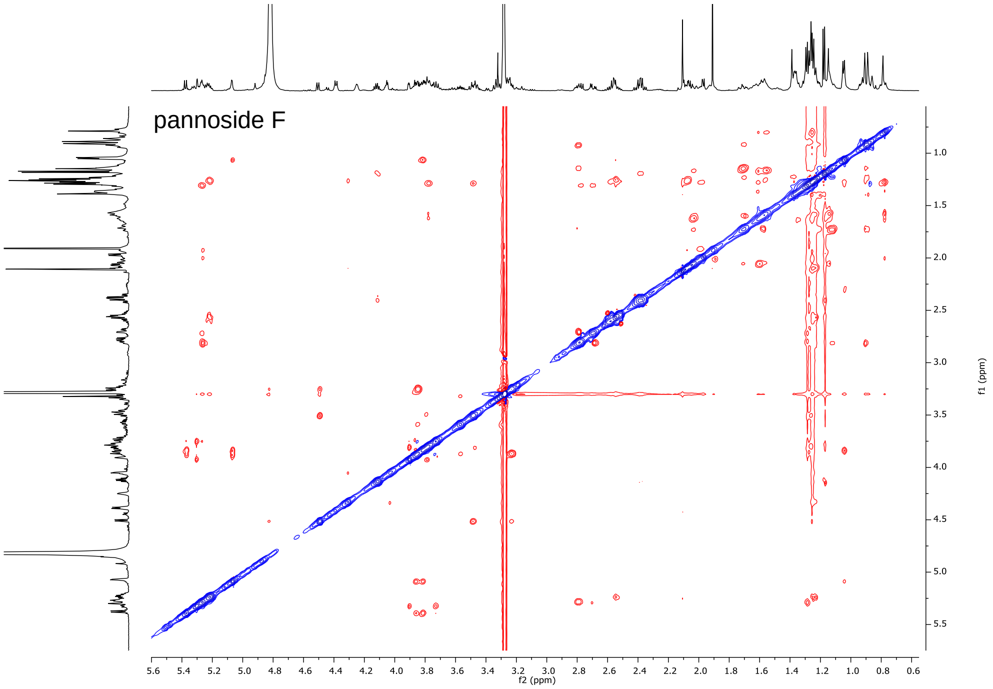

Figure S5. TOCSY NMR spectrum of pannoside F (**1**) in CD_3OD-*d*$_4$.

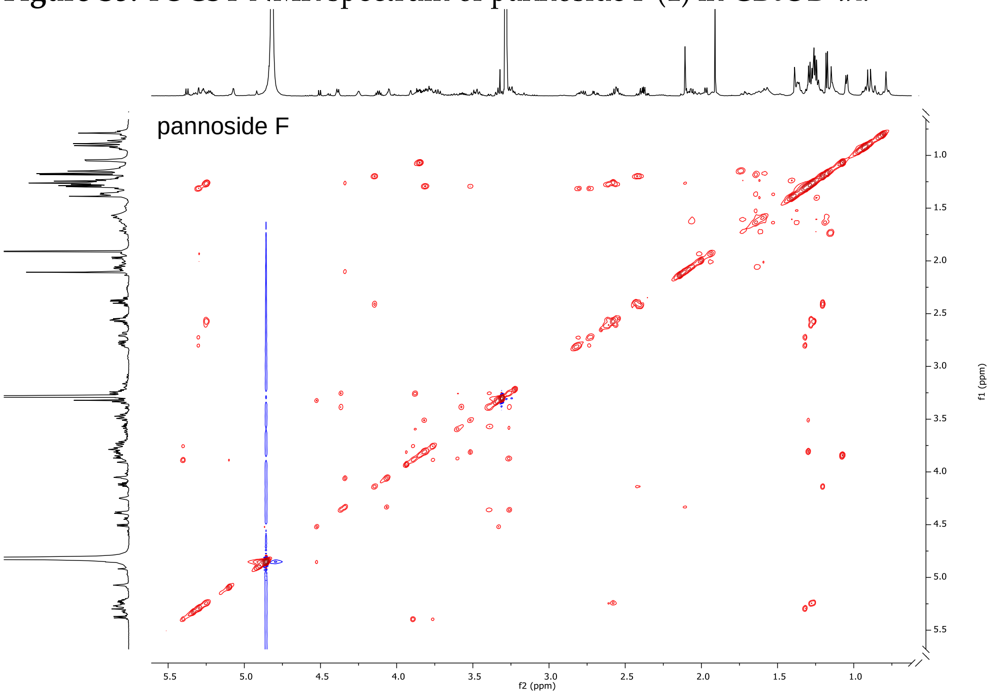

Figure S6. HSQC NMR spectrum of pannoside F (**1**) in CD_3OD-*d*$_4$.

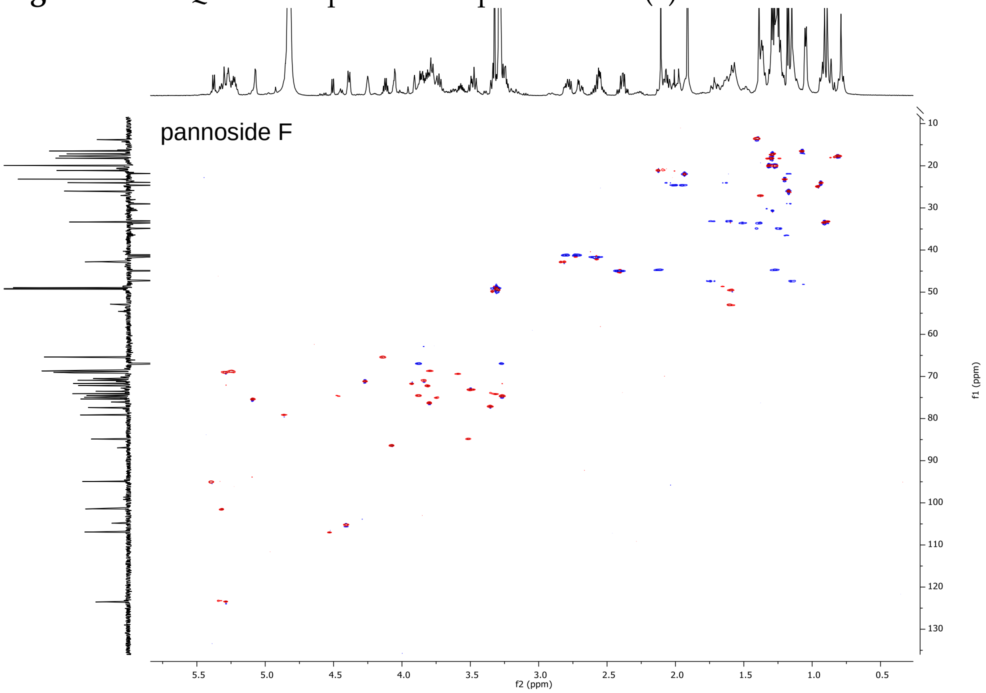

Figure S7. HMBC NMR spectrum of pannoside F (**1**) in CD_3OD-d_4.

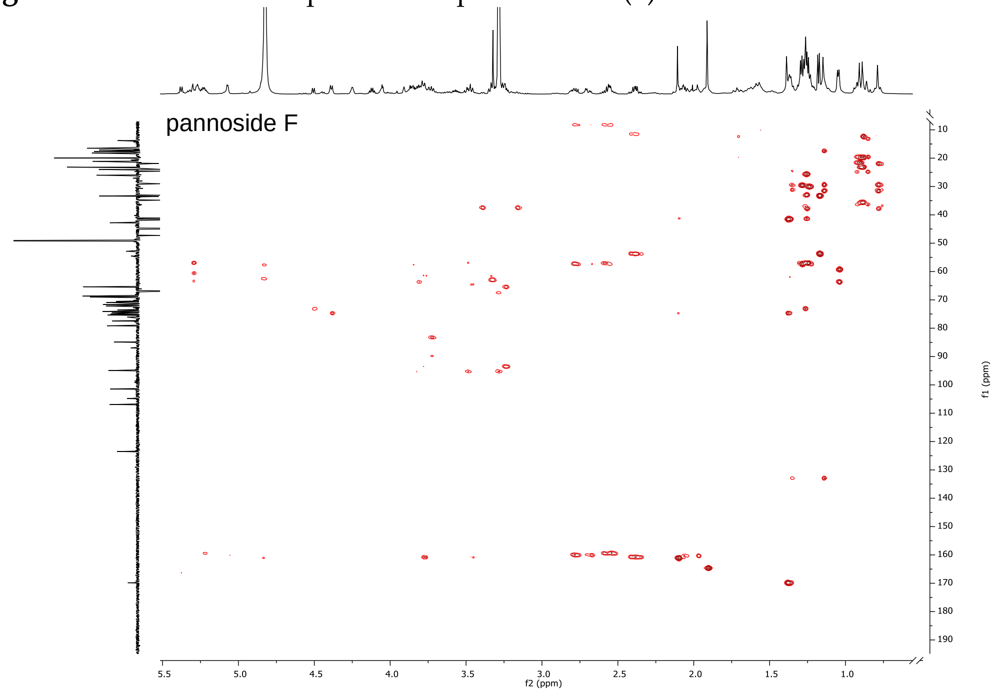

Figure S8. ^{1}H NMR spectrum (600 MHz) of pannoside G (**2**) in CD_3OD-*d*$_4$.

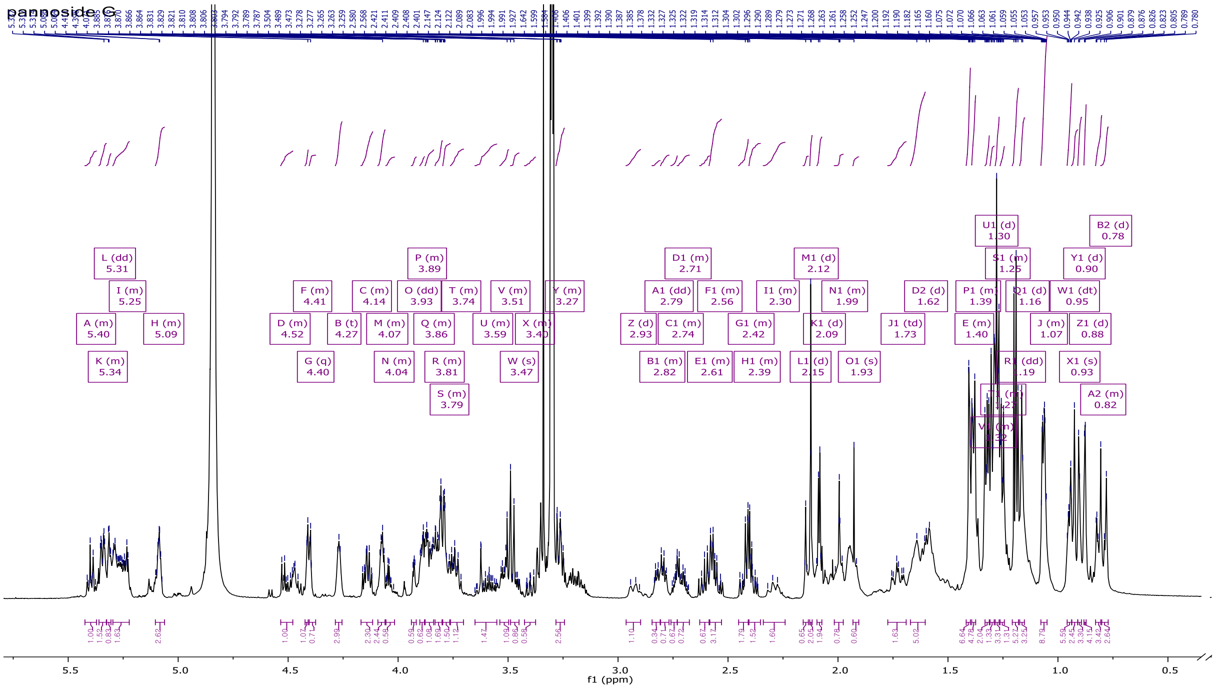

Figure S9. DEPT-135 spectrum (150 MHz) of pannoside G (**2**) in CD_3OD-*d*$_4$.

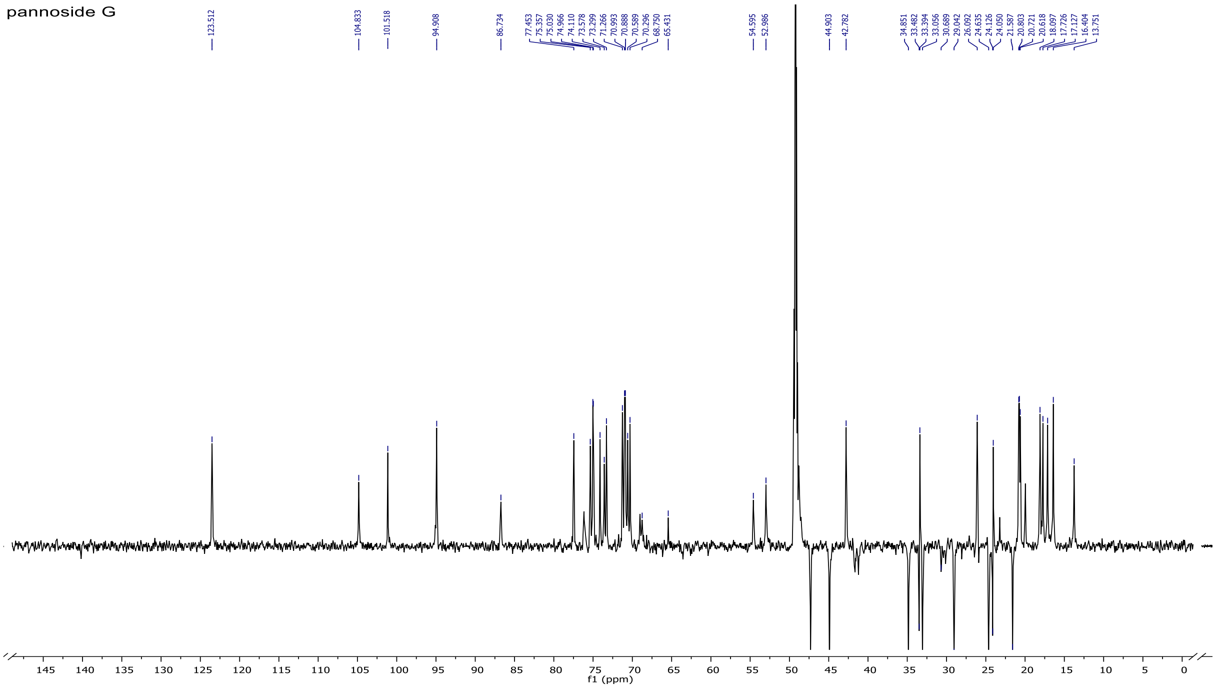

Figure S10. COSY NMR spectrum of pannoside G (**2**) in CD3OD-*d*4.

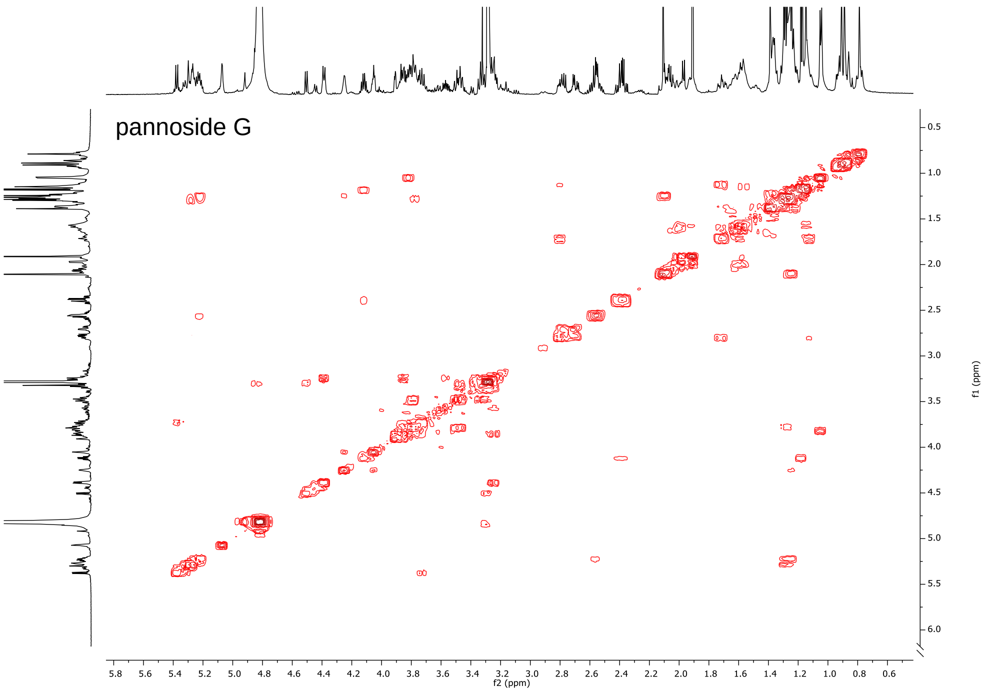

Figure S11. ROESY NMR spectrum of pannoside G (**2**) in CD3OD-*d*4.

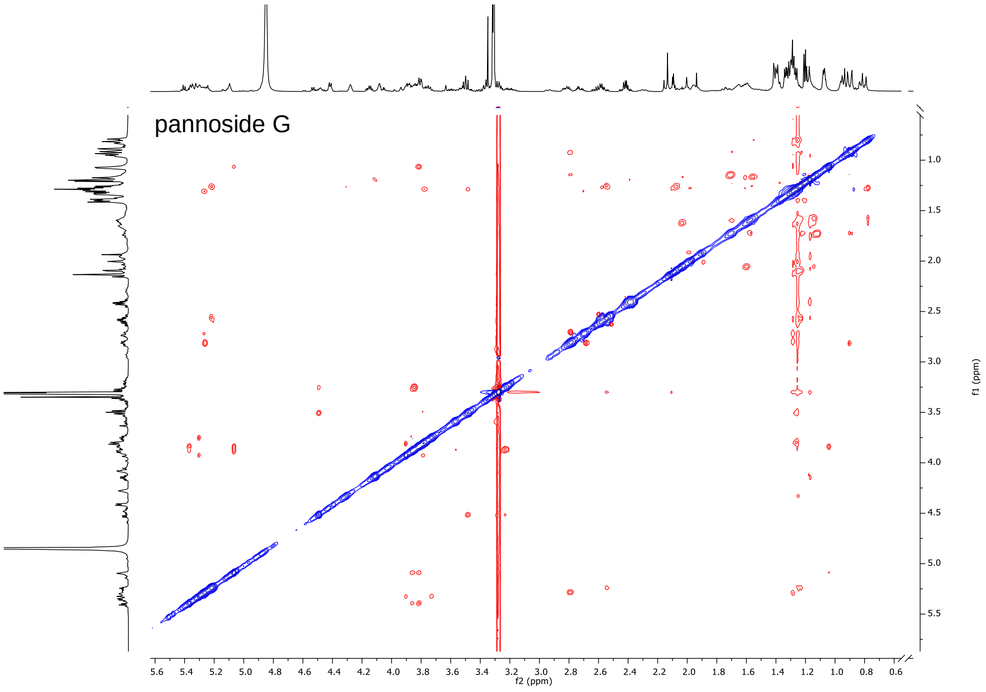

Figure S12. TOCSY NMR spectrum of pannoside G (**2**) in CD_3OD-d_4.

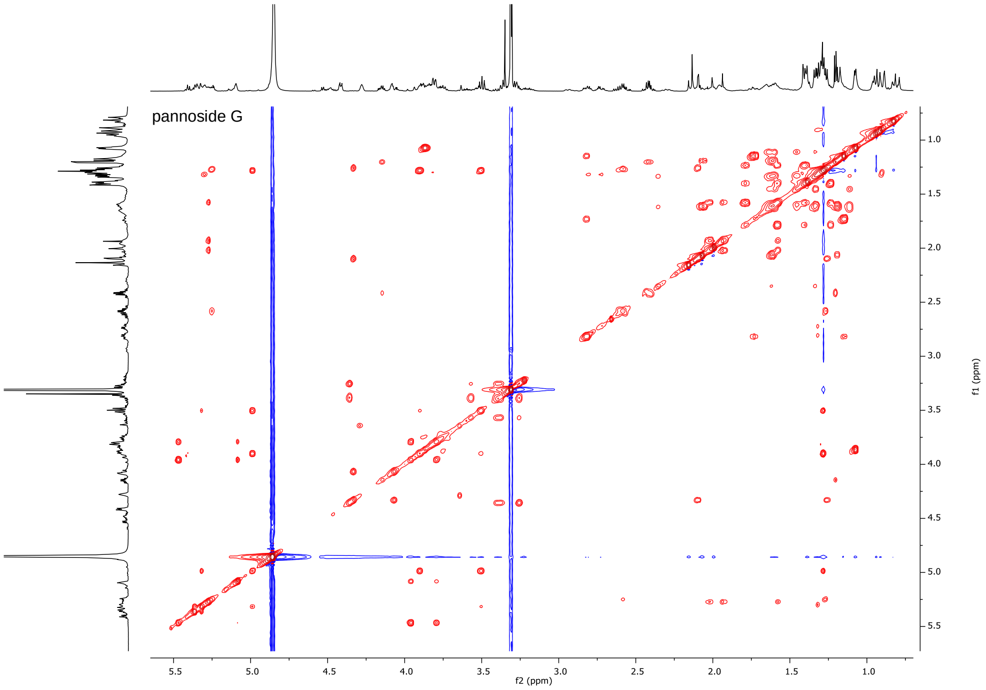

Figure S13. HSQC NMR spectrum of pannoside G (**2**) in CD_3OD-d_4.

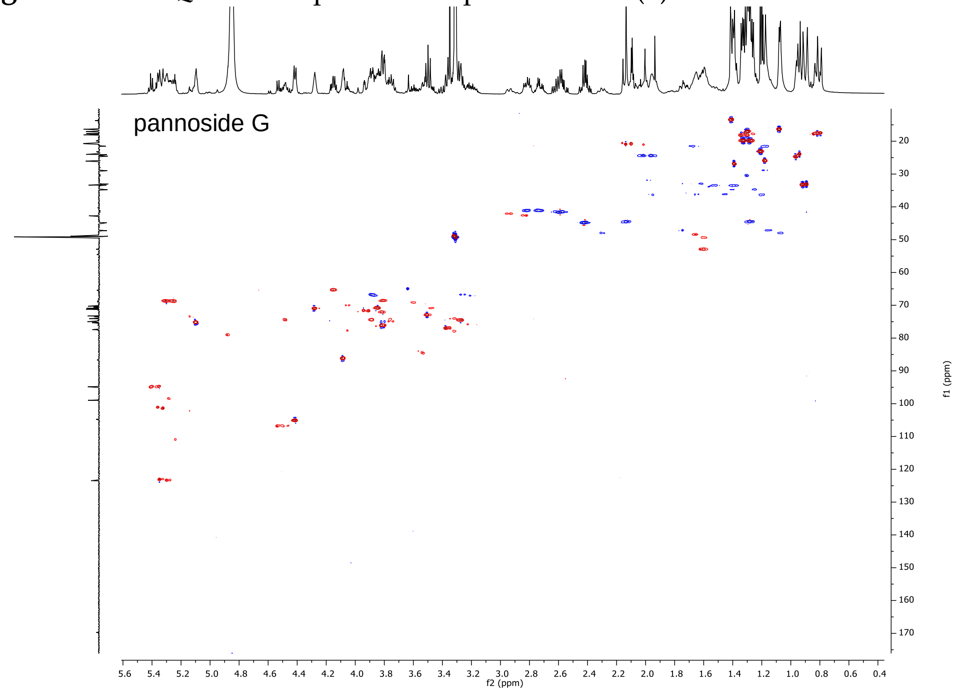

Figure S14. HMBC NMR spectrum of pannoside G (**2**) in CD_3OD-*d*$_4$.

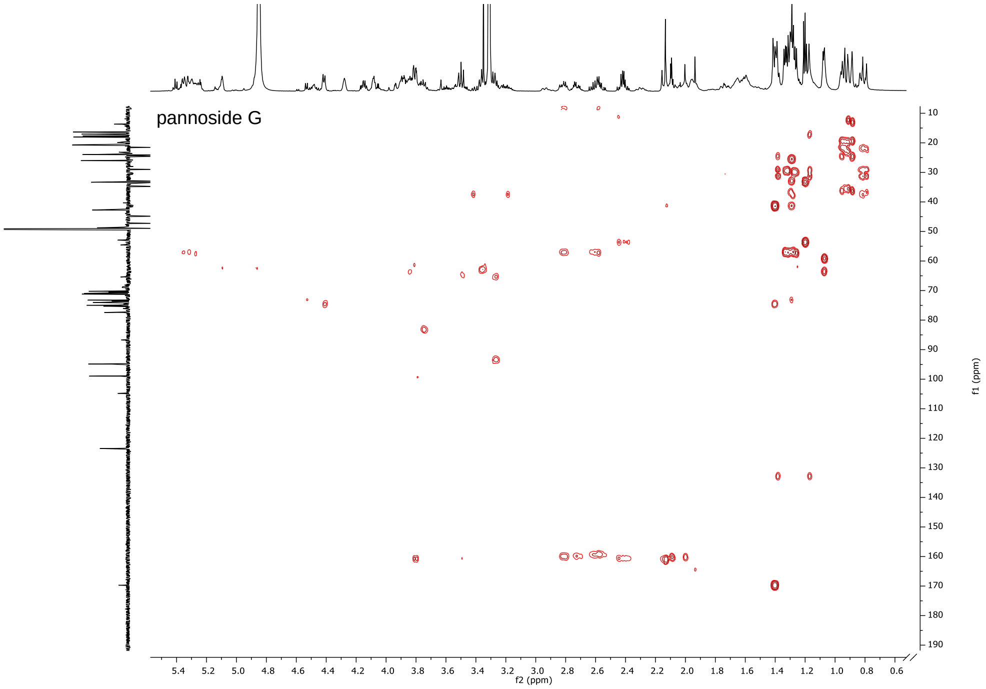

Figure S15. ^{1}H NMR spectrum (600 MHz) of pannoside H (**3**) in CD_3OD-*d*$_4$.

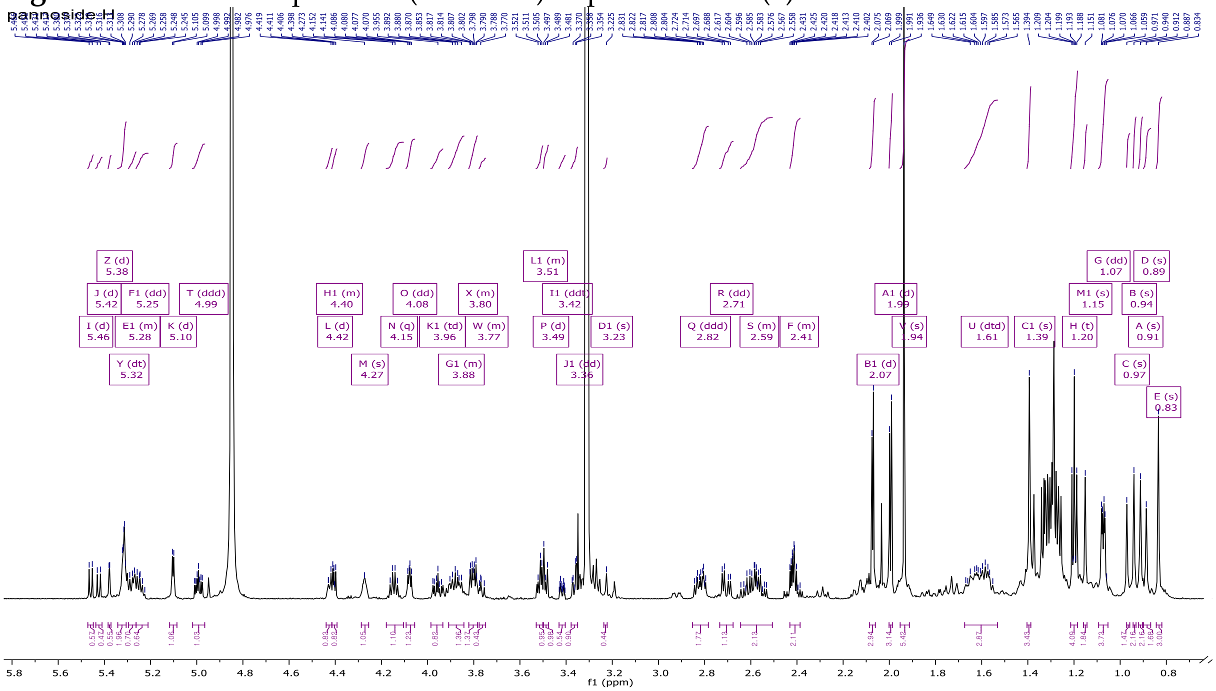

Figure S16. ^{13}C NMR spectrum (150 MHz) of pannoside H (**3**) in CD_3OD-*d*$_4$.

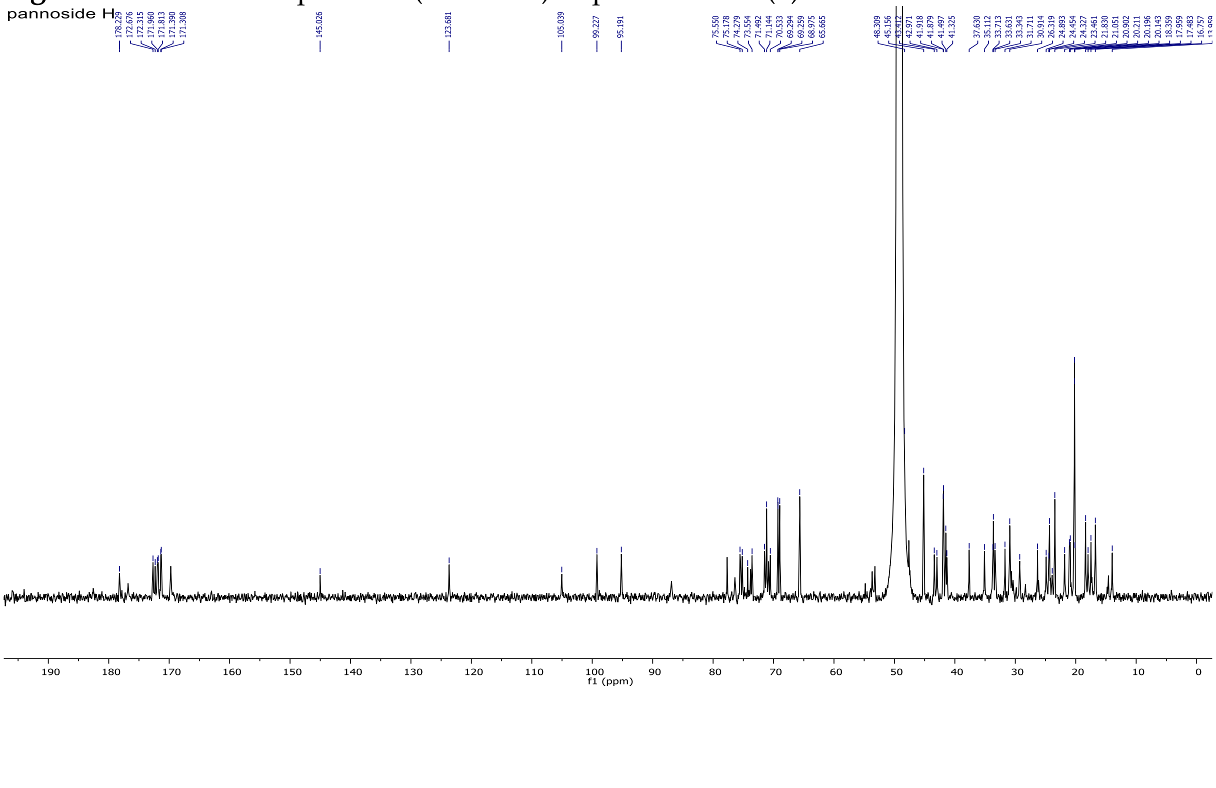

Figure S17. COSY NMR spectrum of pannoside H (**3**) in CD3OD-*d*4.

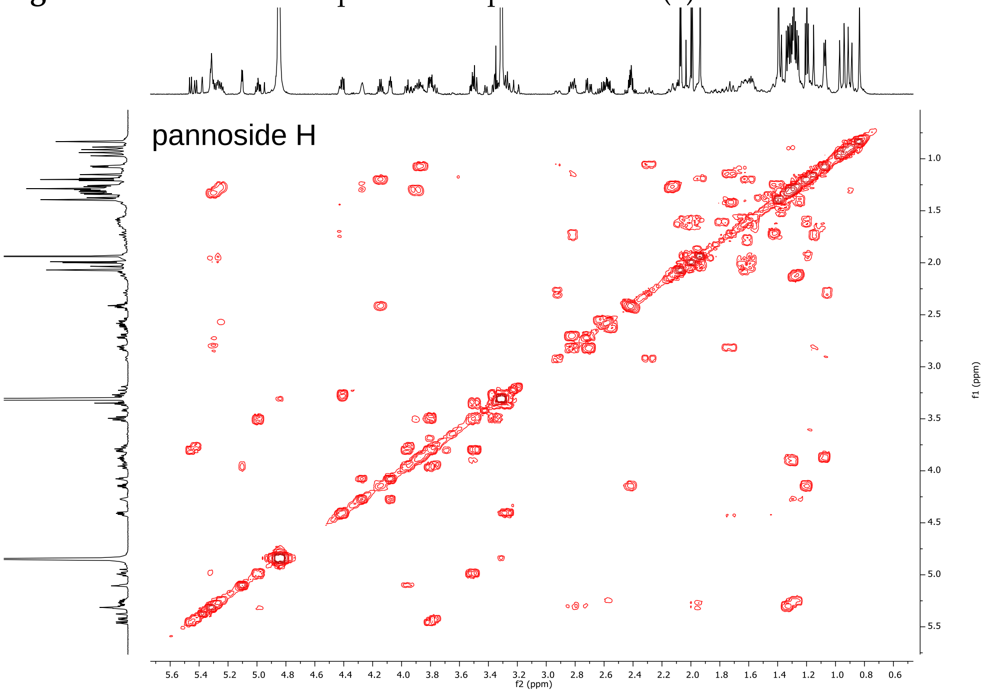

Figure S18. ROESY NMR spectrum of pannoside H (**3**) in CD3OD-*d*4.

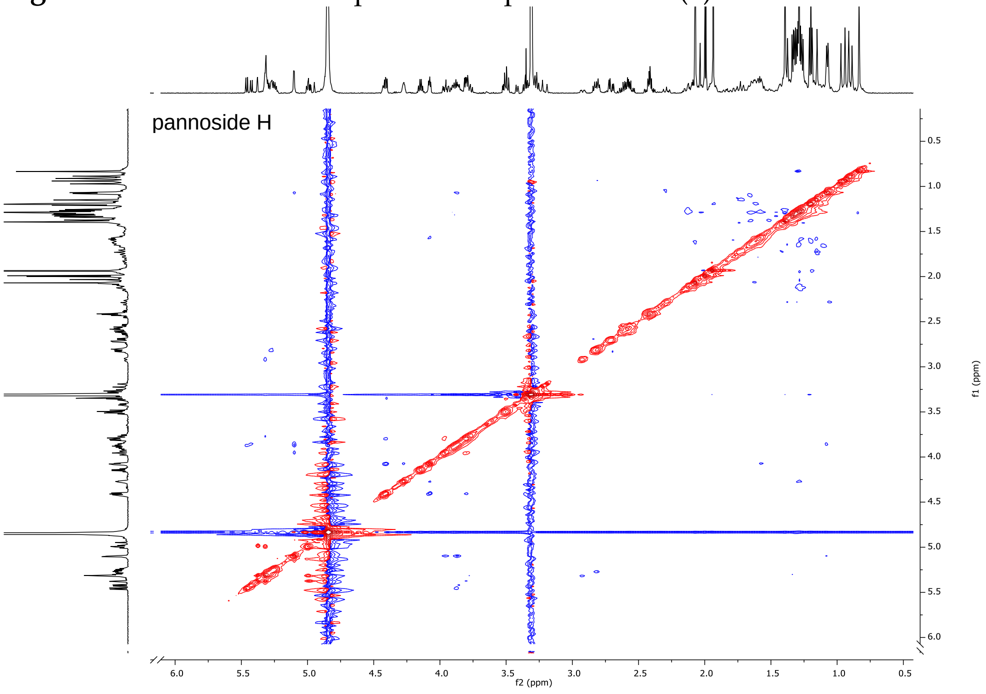

Figure S19. TOCSY NMR spectrum of pannoside H (**3**) in CD_3OD-*d*$_4$.

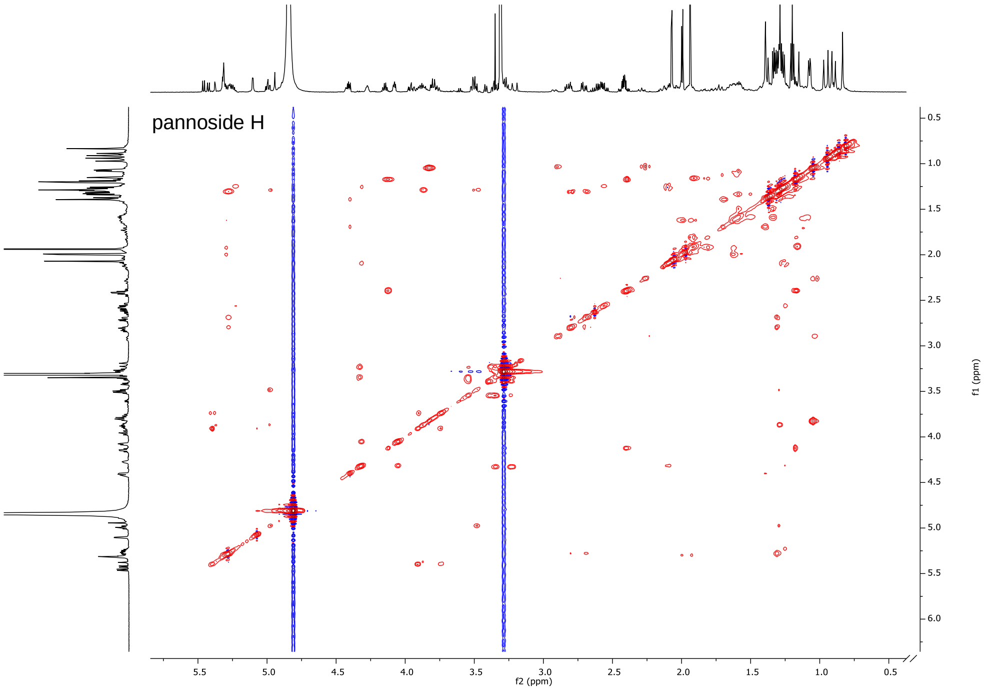

Figure S20. HSQC NMR spectrum of pannoside H (**3**) in CD_3OD-*d*$_4$.

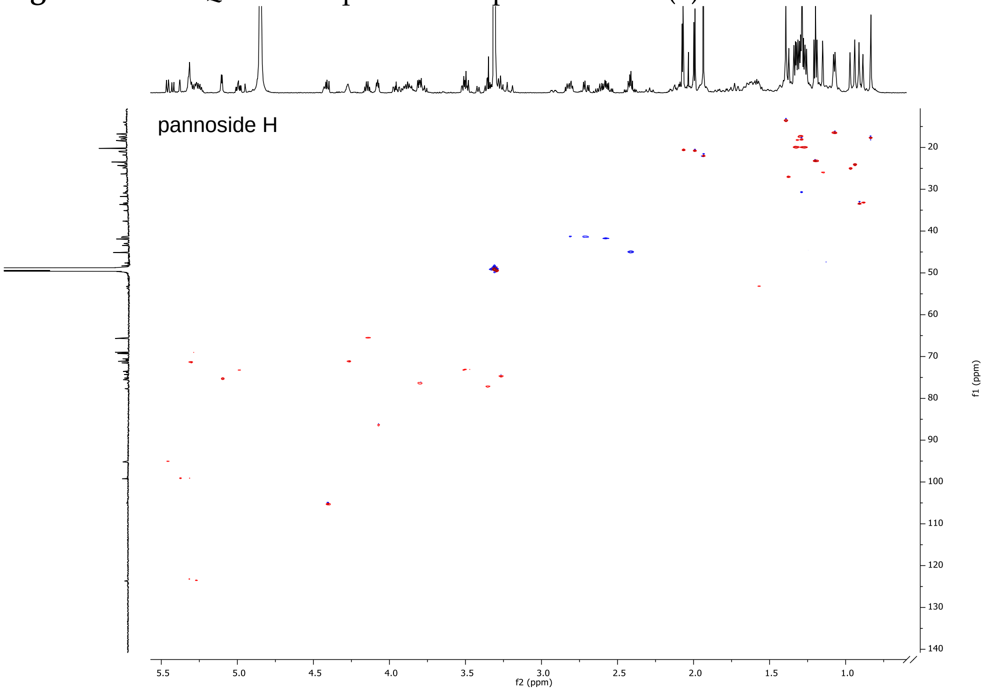

Figure S21. HMBC NMR spectrum of pannoside H (**3**) in CD_3OD-*d*$_4$.

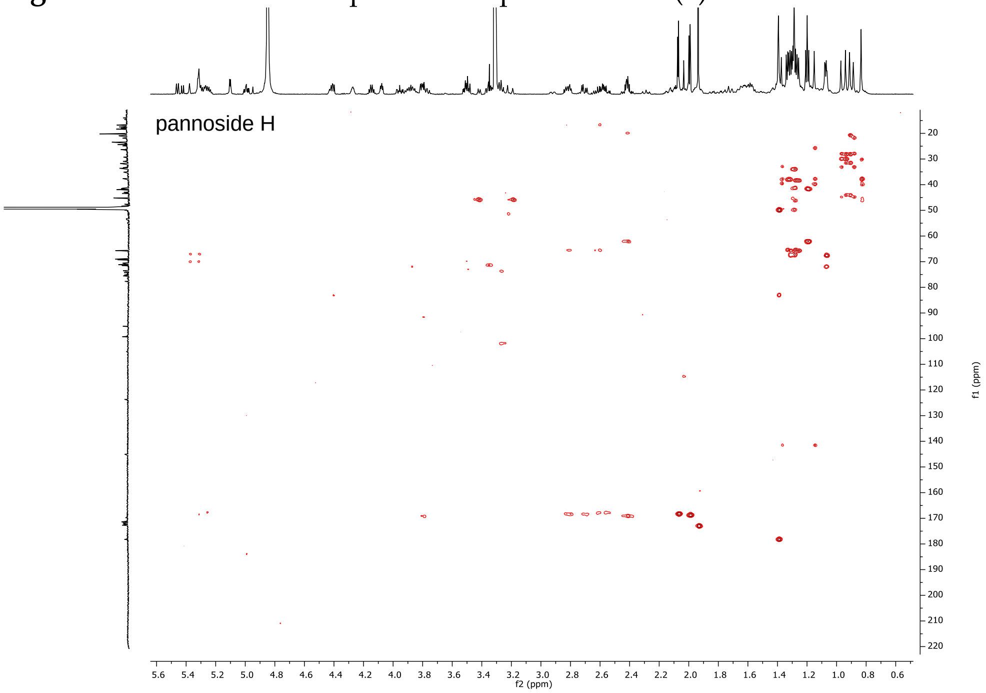

Figure S22. ^{1}H NMR spectrum (600 MHz) of pannoside I (**4**) in CD_3OD-*d*4.

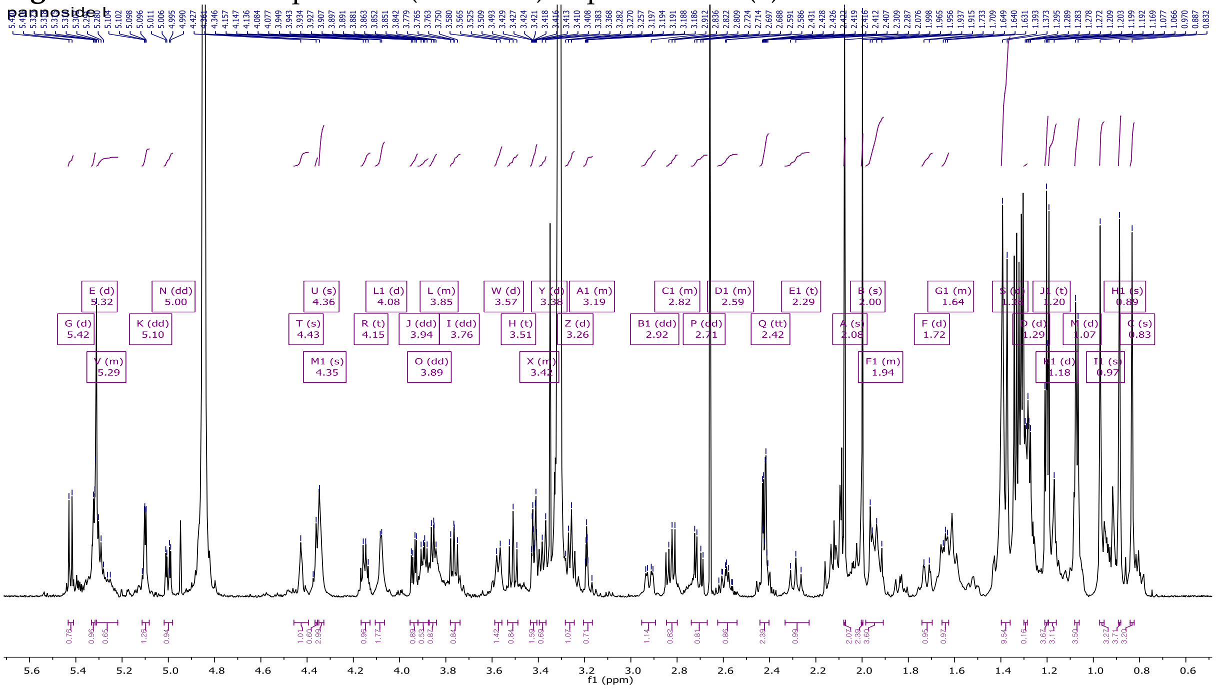

Figure S23. DEPT-135 spectrum (150 MHz) of pannoside I (**4**) in CD_3OD-*d*4.

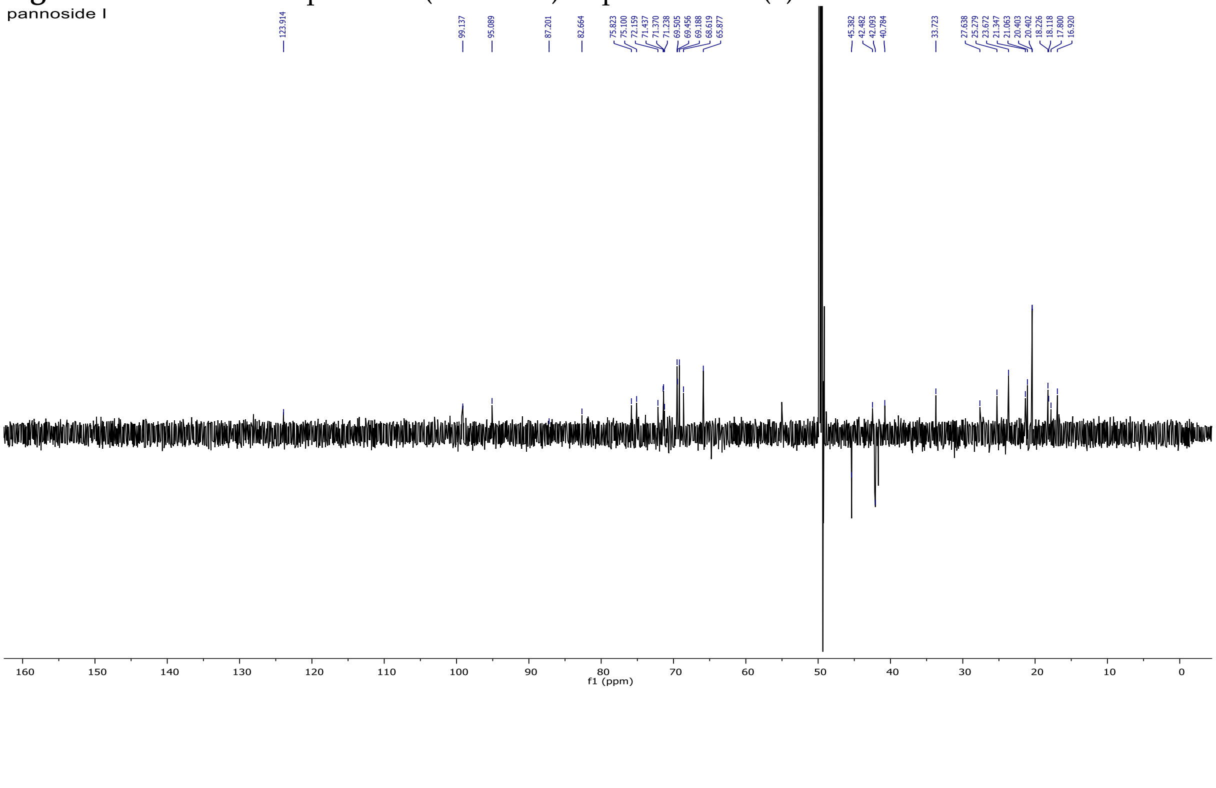

Figure S24. COSY NMR spectrum of pannoside I (**4**) in CD3OD-*d*4.

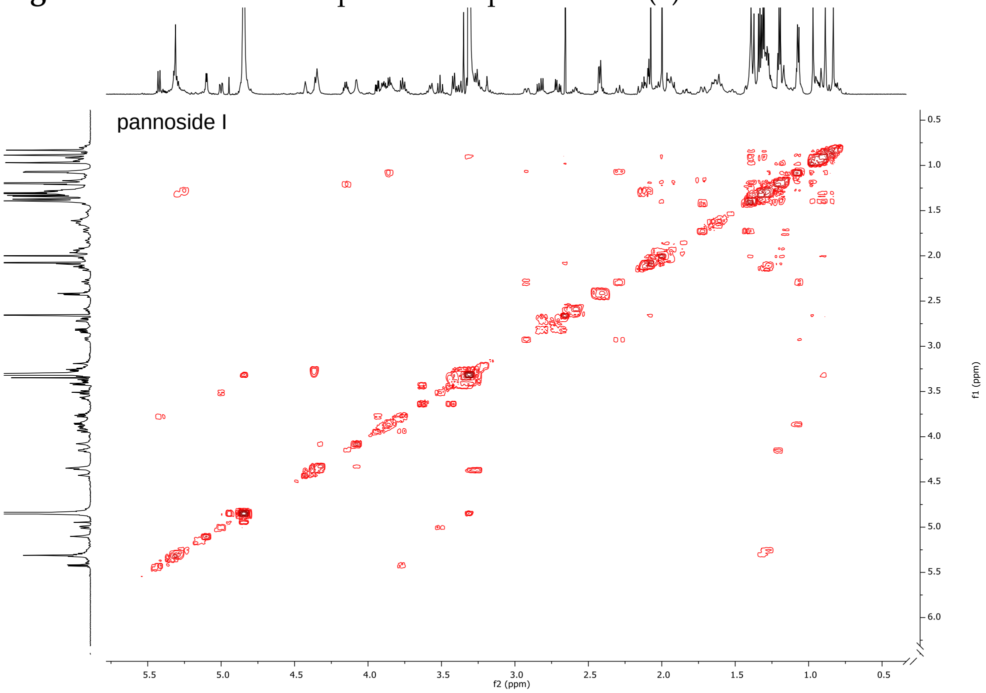

Figure S25. ROESY NMR spectrum of pannoside I (**4**) in CD3OD-*d*4.

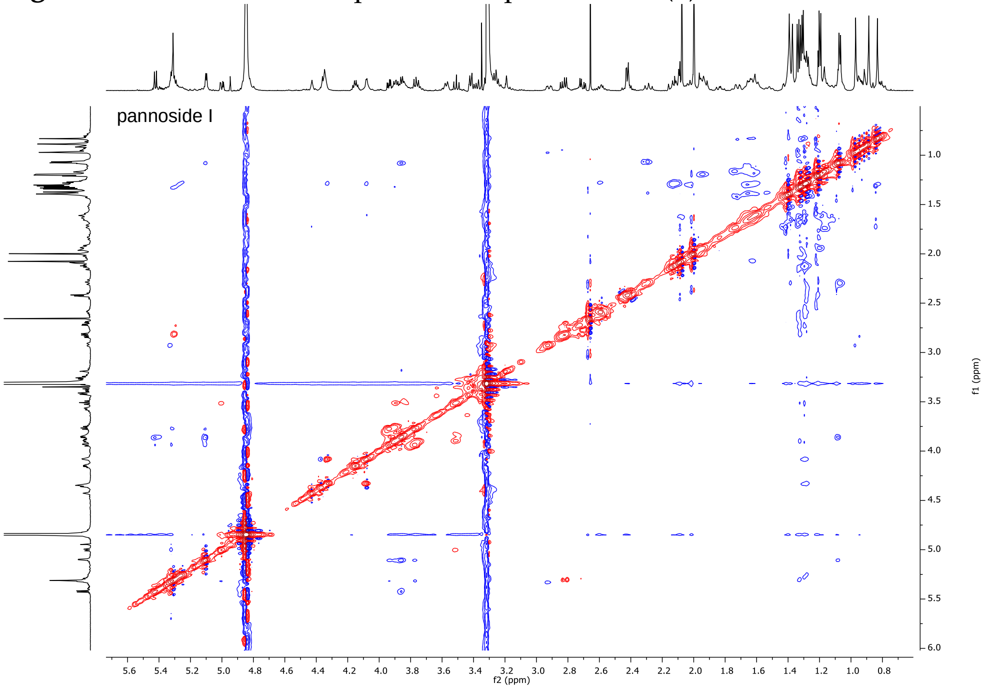

Figure S26. TOCSY NMR spectrum of pannoside I (**4**) in CD_3OD-*d*4.

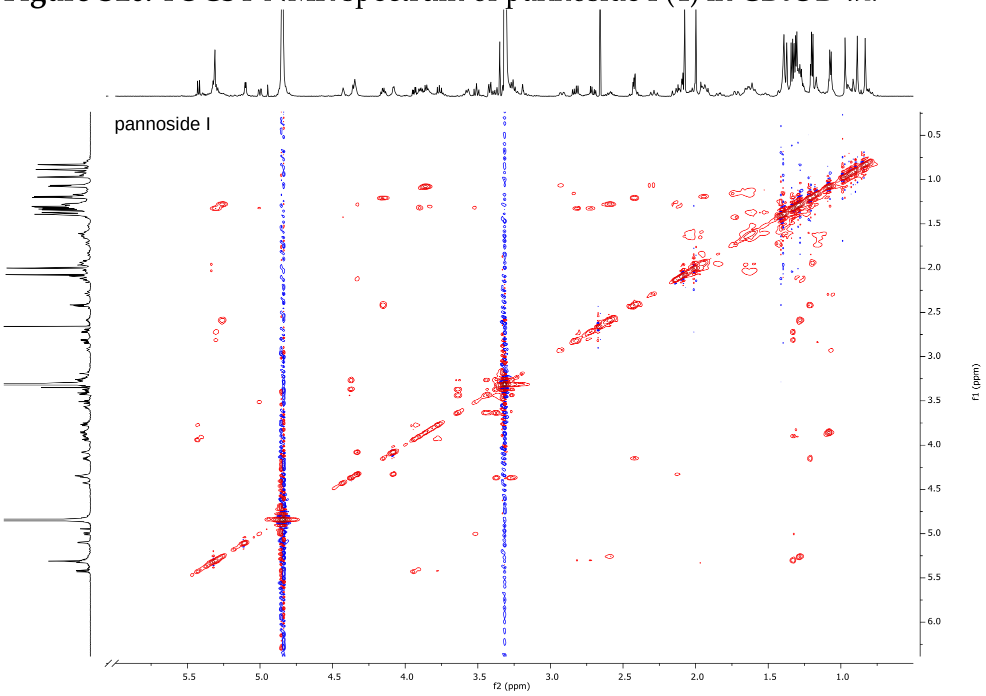

Figure S27. HSQC NMR spectrum of pannoside I (**4**) in CD_3OD-*d*4.

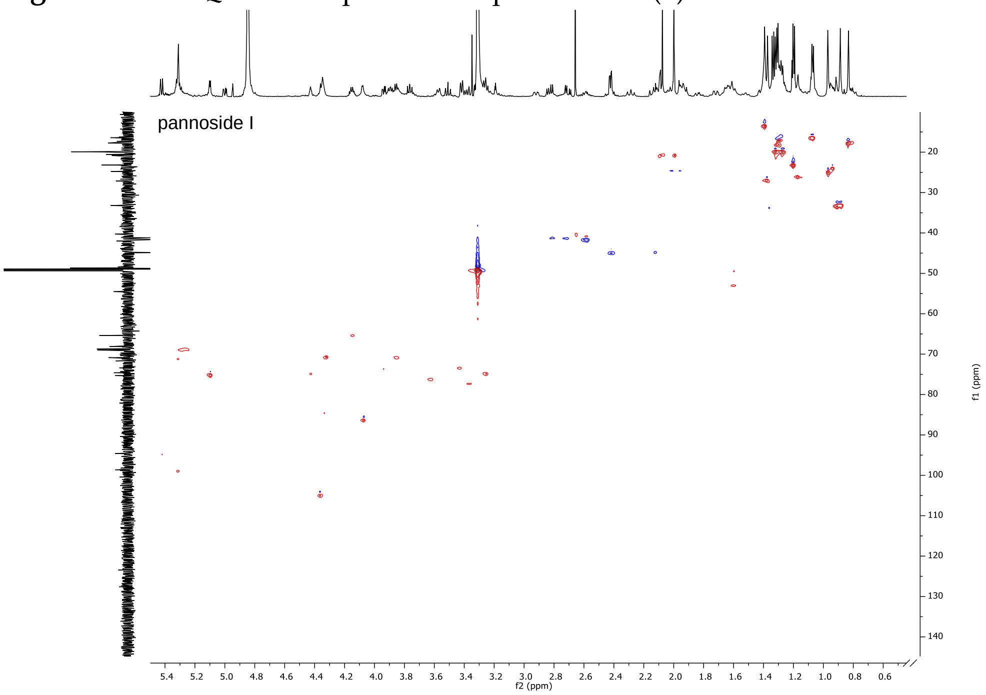

Figure S28. HMBC NMR spectrum of pannoside I (**4**) in CD_3OD-*d*$_4$.

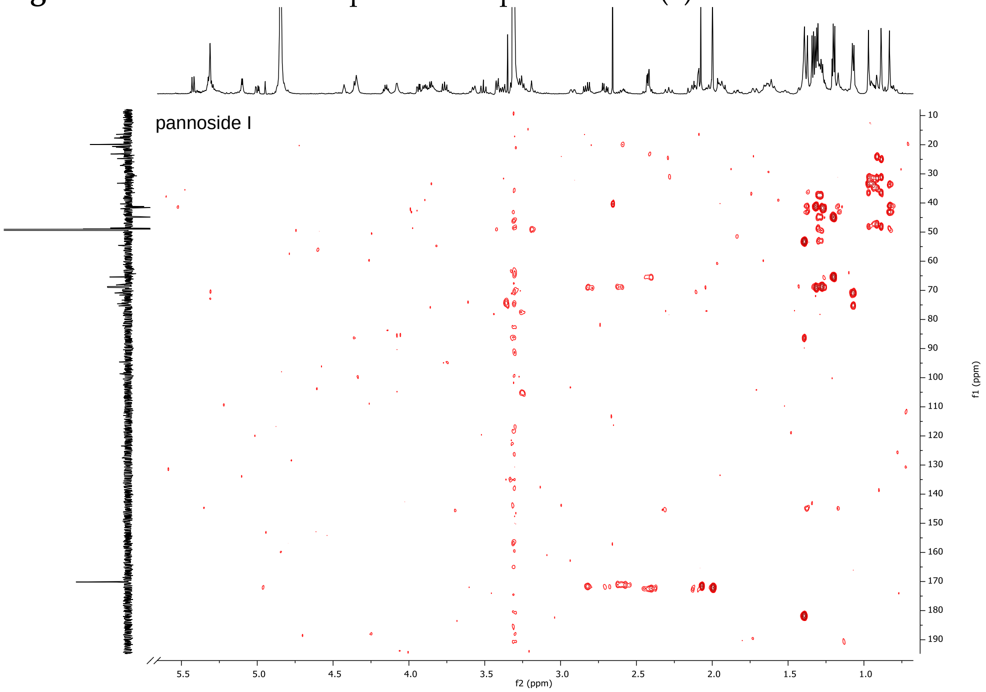

Figure S29. Extracted-ion chromatogram (EIC) of pannosides F–I (**1–4**).

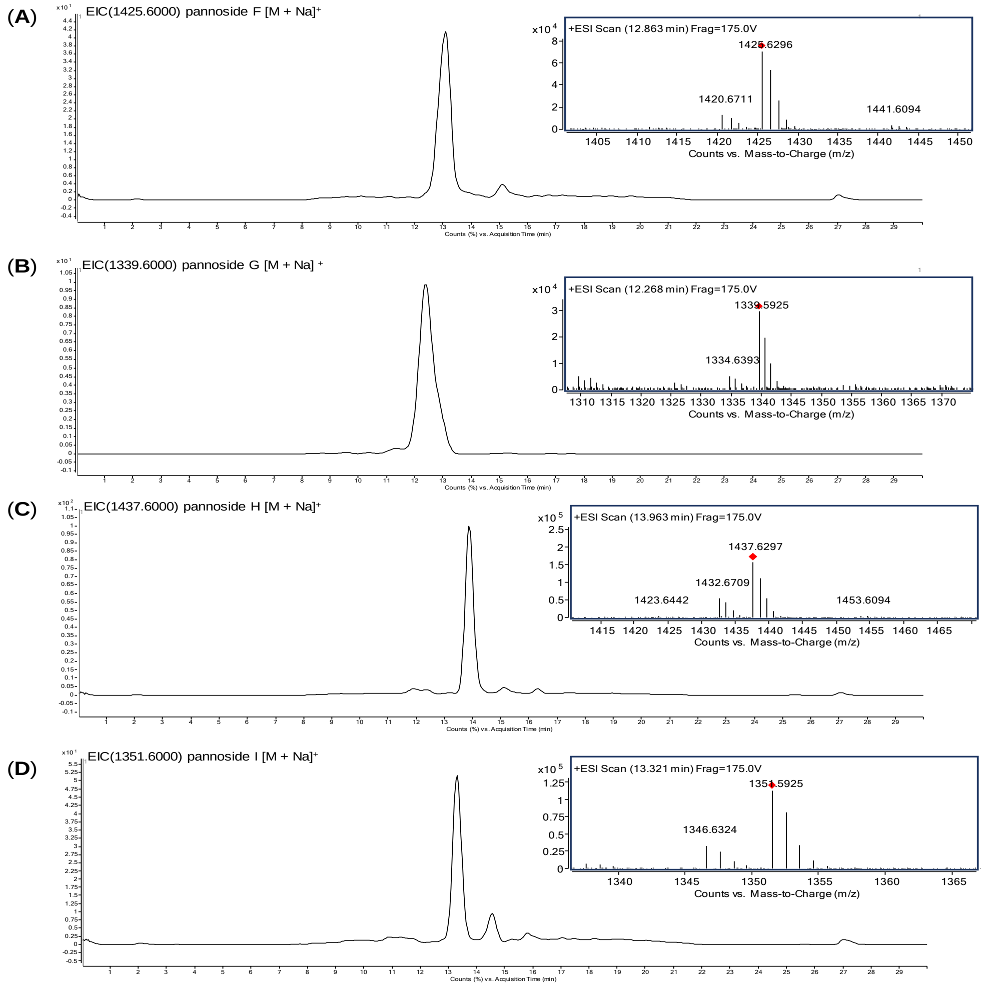

Figure S30. The experimental CD spectra of pannosides F–I (**1–4**).

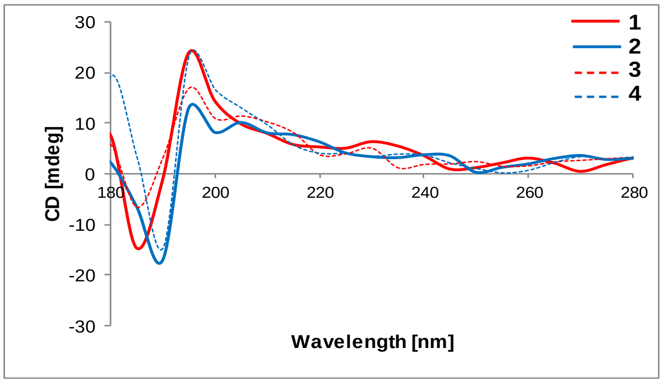

Figure S31. ^{1}H NMR spectrum (600 MHz) of an aglycone of **1** (**1a**) in CD_3OD-*d*$_4$.

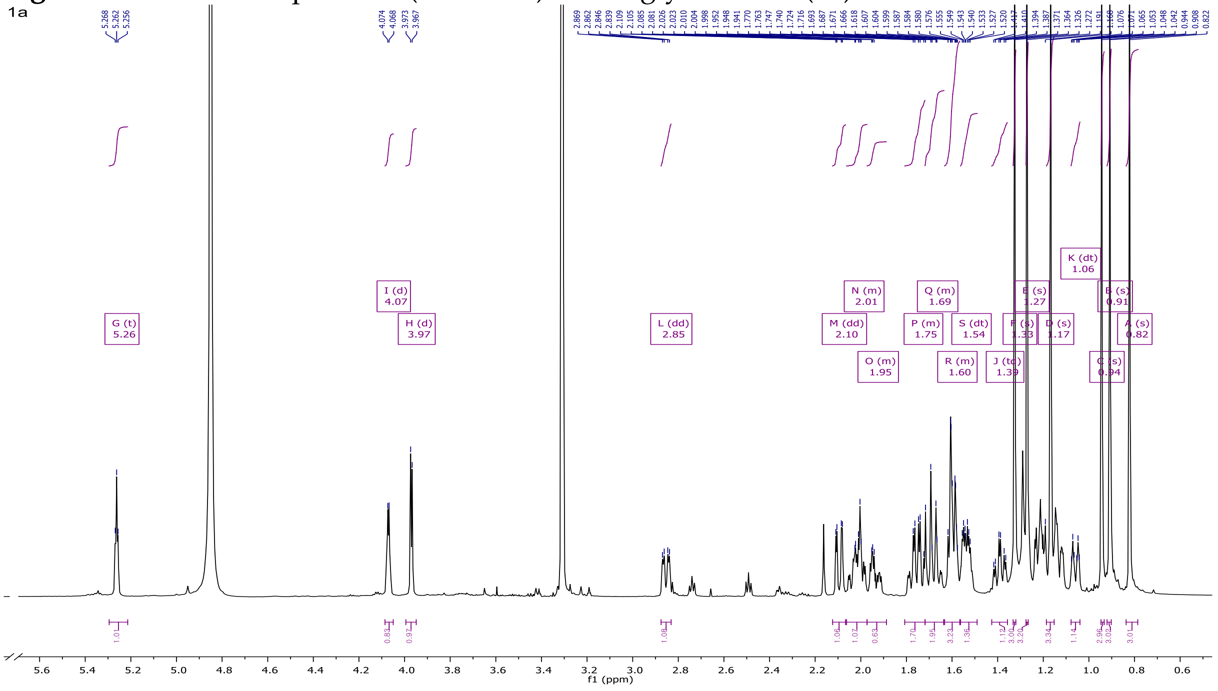

Figure S32. ^{13}C NMR spectrum (150 MHz) of an aglycone of **1** (**1a**) in CD_3OD-*d*$_4$.

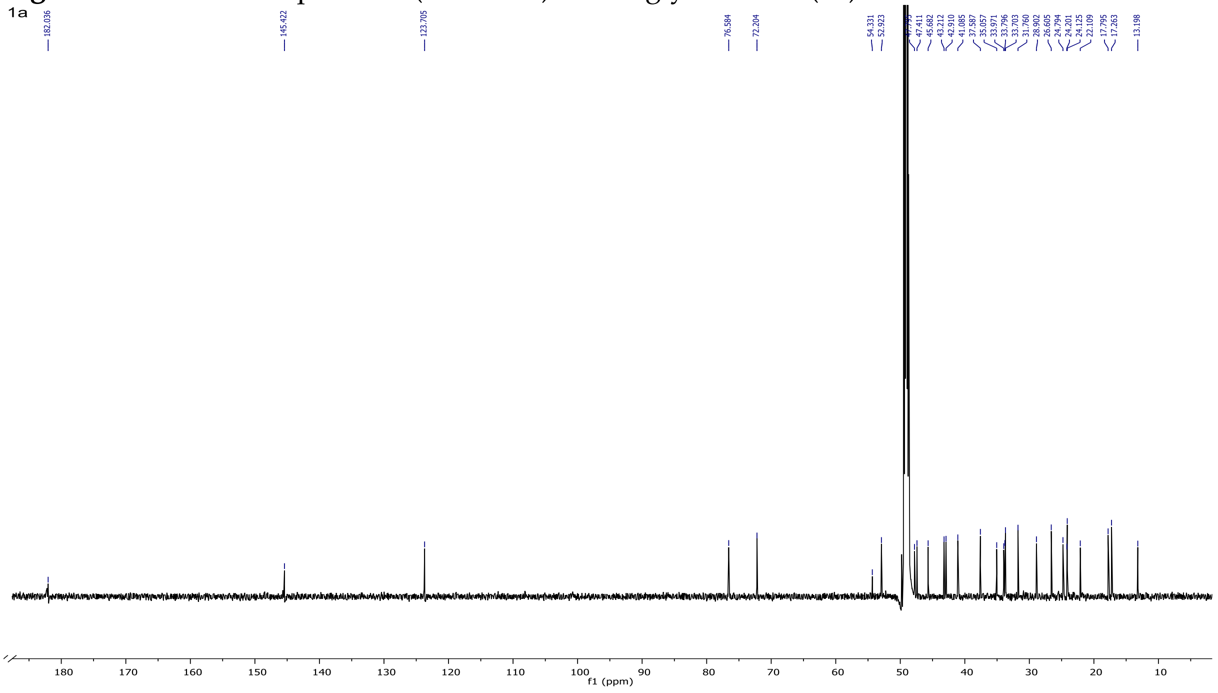

Figure S33. COSY NMR spectrum (400 MHz) of an aglycone of **1** (**1a**) in CD_3OD-*d*4.

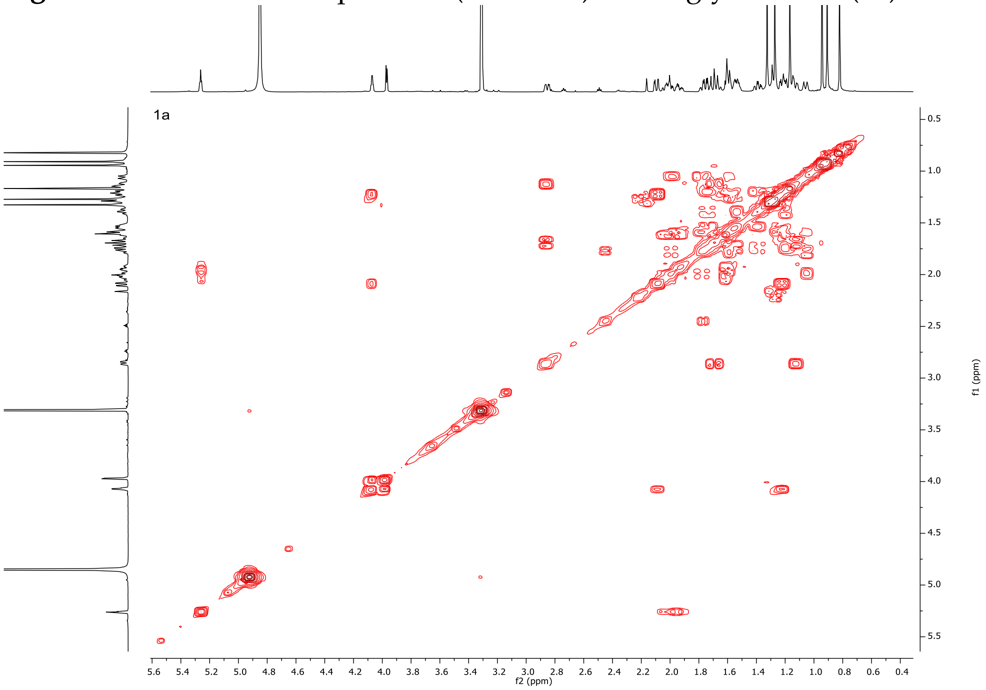

Figure S34. ROESY NMR spectrum (400 MHz) of an aglycone of **1** (**1a**) in CD_3OD-*d*4.

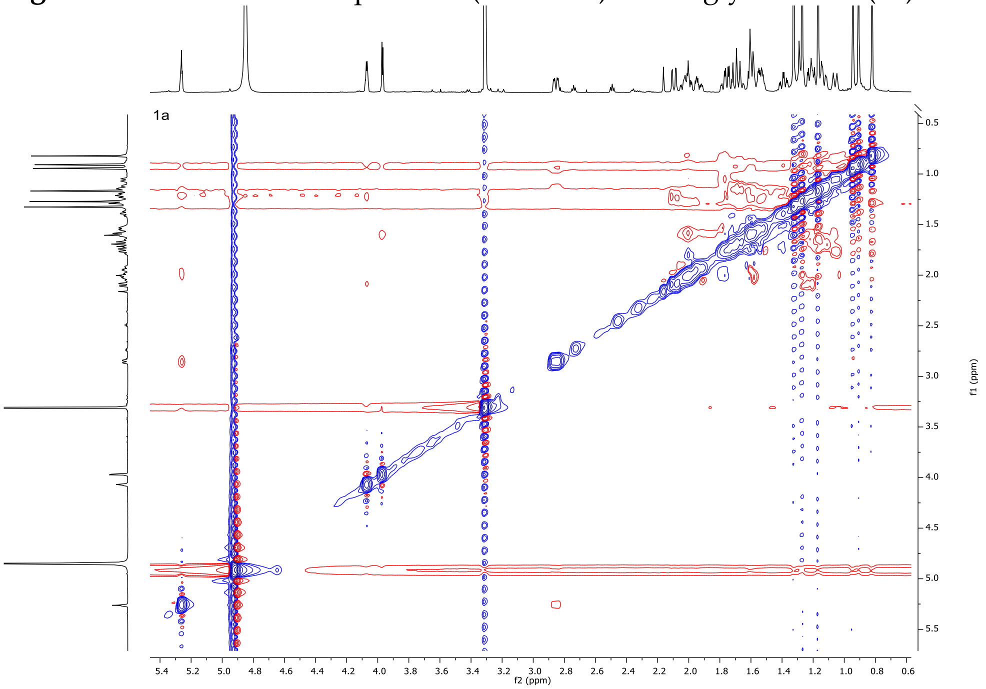

Figure S35. ^{1}H NMR spectrum (400 MHz) of an aglycone of **3** (**3a**) in CD_3OD-d_4.

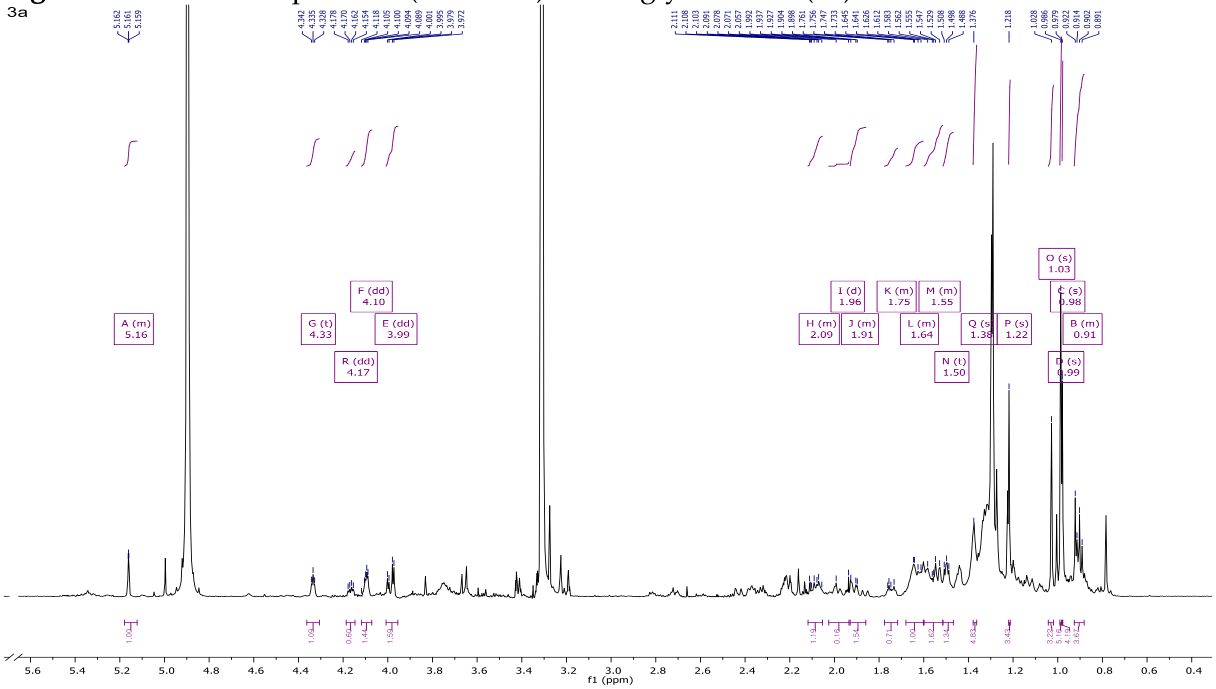

Figure S36. COSY NMR spectrum of an aglycone of **3** (**3a**) in CD_3OD-d_4.

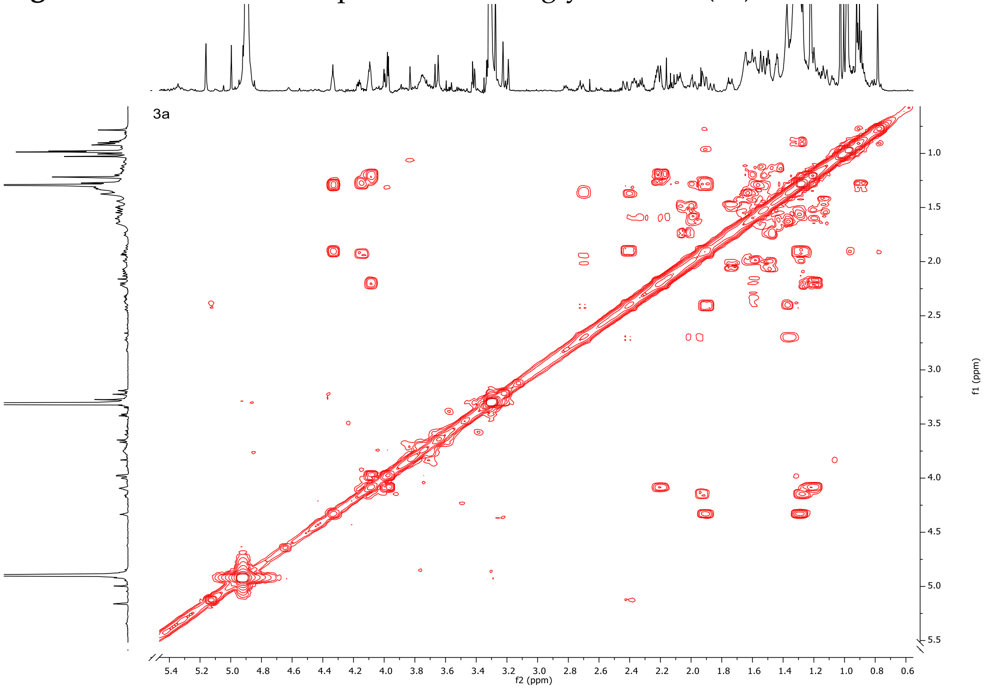

Figure S37. ROESY NMR spectrum of an aglycone of **3** (**3a**) in CD_3OD-d_4.

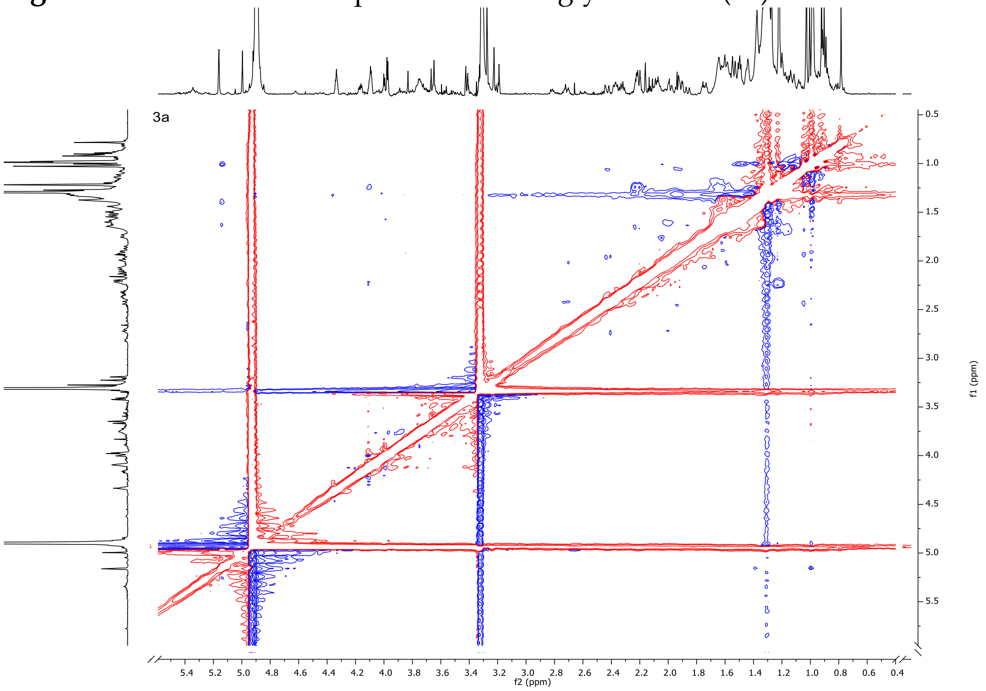

Figure S38. Extracted-ion chromatograms (EIC) of **1a** and **3a**.

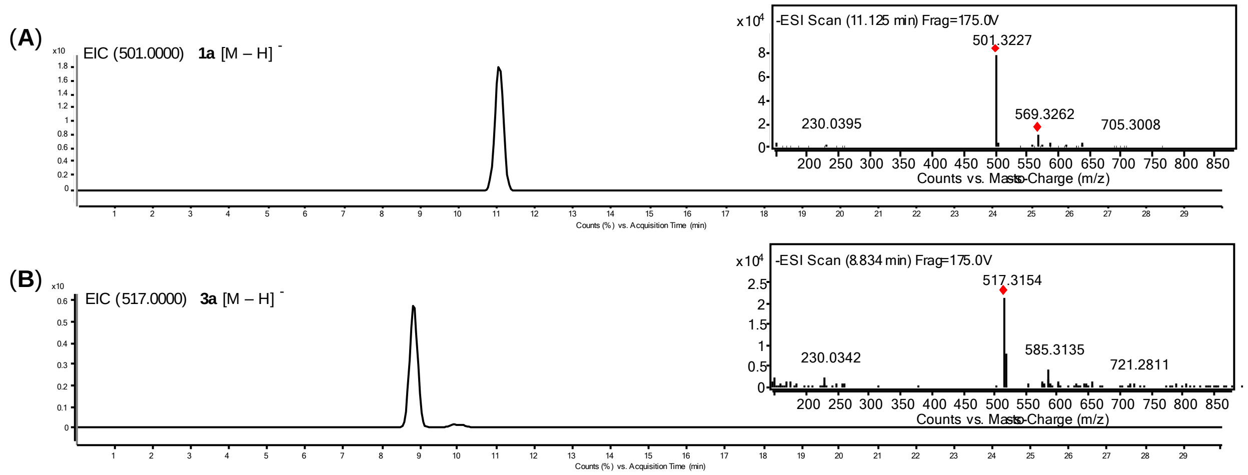

Figure S39. EIC of *S*-PGME derivatives; authentic (*S*)-, (*R*)-3-HB and 3-HB residues in hydrolysate of **1**.

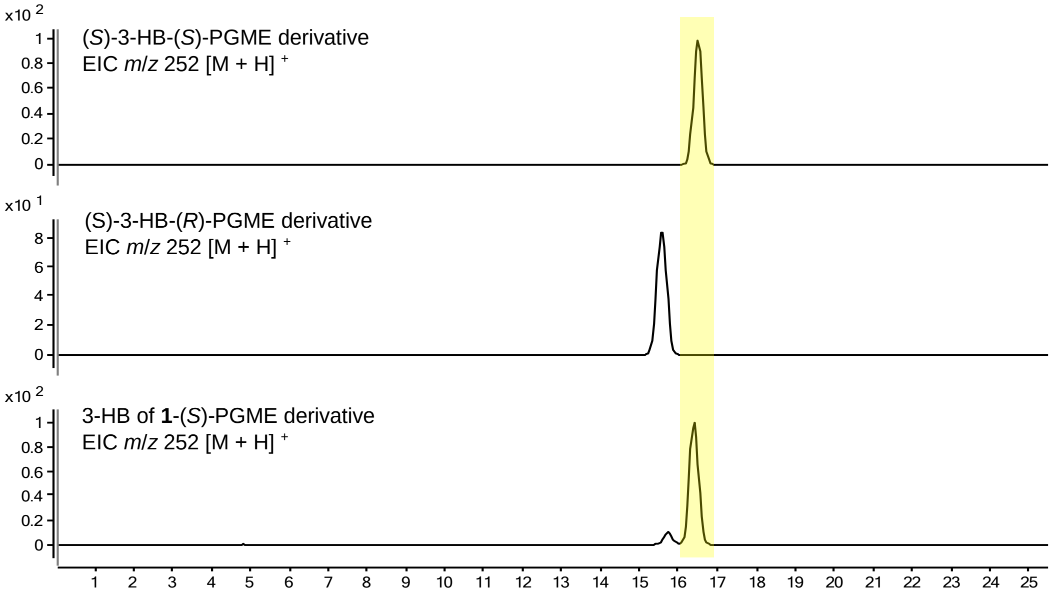

Supplement: Supplementary file 1 [file marinedrugs-22-00524-s001.zip › marinedrugs-3298221-supplementary.pdf]
